# Bioactive Ascochlorin Analogues from the Marine-Derived Fungus *Stilbella fimetaria*

**DOI:** 10.3390/md19020046

**Published:** 2021-01-20

**Authors:** Karolina Subko, Sara Kildgaard, Francisca Vicente, Fernando Reyes, Olga Genilloud, Thomas O. Larsen

**Affiliations:** 1 DTU Bioengineering, Technical University of Denmark, Søltofts Plads 221, DK-2800 Kgs. Lyngby, Denmark; karosu@dtu.dk; 2 Department of Biology, Universitetsparken 15, DK-2100 København Ø, Denmark; sara.kildgaard@bio.ku.dk; 3 Fundación MEDINA, Avda. del Conocimiento, 34, Armilla, 18016 Granada, Spain; francisca.vicente@medinaandalucia.es (F.V.); fernando.reyes@medinaandalucia.es (F.R.); olga.genilloud@medinaandalucia.es (O.G.)

**Keywords:** meroterpenoids, ascochlorin, bioactivity, dereplication, MS/HRMS

## Abstract

The marine-derived fungus *Stilbella fimetaria* is a chemically talented fungus producing several classes of bioactive metabolites, including meroterpenoids of the ascochlorin family. The targeted dereplication of fungal extracts by UHPLC-DAD-QTOF-MS revealed the presence of several new along with multiple known ascochlorin analogues (**19**–**22**). Their structures and relative configuration were characterized by 1D and 2D NMR. Further targeted dereplication based on a novel 1,4-benzoquinone sesquiterpene derivative, fimetarin A (**22**), resulted in the identification of three additional fimetarin analogues, fimetarins B–D (**23**–**25**), with their tentative structures proposed from detailed MS/HRMS analysis. In total, four new and eight known ascochlorin/fimetarin analogues were tested for their antimicrobial activity, identifying the analogues with a 5-chloroorcylaldehyde moiety to be more active than the benzoquinone analogue. Additionally, the presence of two conjugated double bonds at C-2′/C-3′ and C-4′/C-5′ were found to be essential for the observed antifungal activity, whereas the single, untailored bonds at C-4′/C-5′ and C-8′/C-9′ were suggested to be necessary for the observed antibacterial activity.

## 1. Introduction

**Figure 1:**
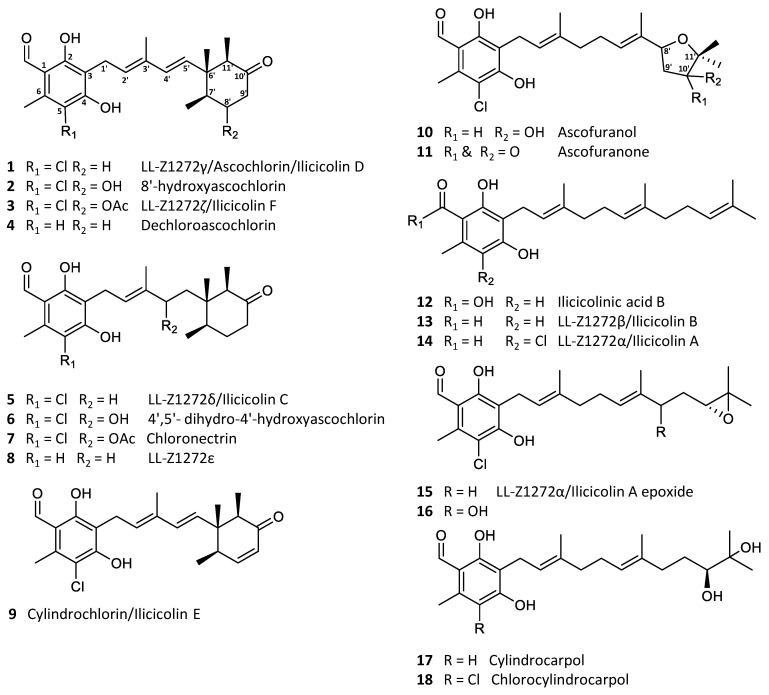
Examples of the most common ascochlorin analogues.

Ascochlorin (**1**) is a meroterpenoid, comprised of 5-chloroorcylaldehyde with C-3 substitution of a C6′–C11′ cyclized sesquiterpene side chain [1]. Ascochlorin was first isolated from a soil isolate *Ascochyta viciae* (later re-identified as *Acremonium sclerotigenum*) in 1968 [2,3], with the absolute structure determined by X-ray crystallography a year later [4]. Subsequently, numerous related analogues have been isolated from the original fungus [5,6], as well as several other fungi within the Hypocreales order, such as *Cylindrocladium ilicicola* (=*Calonectria pyrochroa*) [7], *Nectria galligena* (=*Neonectria ditissima*) [8], *Fusarium* sp. [9] of terrestrial origin, and sponge-derived *Acremonium* sp. [10] and *Stachybotrys* sp. [11] of marine origin. The majority of the analogues constitute simple backbone modifications, such as halogenation, dehydrogenation, hydroxylation, as well as acetylation of an alcohol group (**2**–**9**), with alternative sesquiterpene cyclization to form an oxygen-containing heterocycle between 8′OH and C-11′ (**10**–**11**) or a linear sesquiterpene chain being the minor analogues (**12**–**17**; Figure 1) [5,9,10,12,13]. Furthermore, similar compounds with a single isoprene or monoterpene side chain have also been reported [8,14,15,16]. 

Ascochlorin derivatives were originally known for their antibacterial [17], anticancer, and antiviral activities [1,2,18]. Additionally, a series of physiological modulation activities have also been reported, such as anti-inflammatory [19,20], hypolipidemic [5], and antidiabetic activities [21,22]. Notably, ascofuranone (**11**) is a promising candidate against the neglected tropical disease, African trypanosomiasis [23,24]. The biosynthesis of ascochlorin (**1**) and ascofuranone (**11**) in *Acremonium egyptiacum* (=*Acremonium sclerotigenum*) [25] has recently been elucidated, which has made it possible to specifically target the production of desired bioactive analogues [26,27]. Interestingly, the biosynthesis of both **1** and **11** starts by farnesylation of orsellinic acid to form the meroterpenoid **12,** which upon further modifications result in **13**, **14**, and **15**. The ilicicolin A epoxide (**15**) is the point of divergence, where **1** is produced through intermediate **5**, and **11** through intermediates **16** and **10** (Figure 1). Therefore, by targeted knockout, selective production of either **1** or **11** can be achieved [27]. 

Recently, the marine-derived fungus *Stilbella fimetaria* was shown to be a chemically talented fungus, producing several classes of bioactive compounds, such as antibacterial peptaibiotics, antifungal PKS-NRPS hybrids of the ilicicolin H family, and cytotoxic pimarane-type diterpenoids [28]. In this study we follow up on our previous *Stilbella fimetaria* investigation with ascochlorin and related analogue analysis by ultra-high performance liquid chromatography diode array detection quadrupole time of flight mass spectrometry (UHPLC-DAD-QTOF-MS). In particular, we report the discovery of a novel 1,4-benzoquinone sesquiterpene derivative, fimetarin A, along with MS/HRMS-based tentative identification of three additional analogues, fimetarins B–D. The antimicrobial activities of all isolated compounds, including fimetarin A, three new, and eight known ascochlorin analogues, were evaluated. 

## 2. Results and Discussion

**Figure 2:**
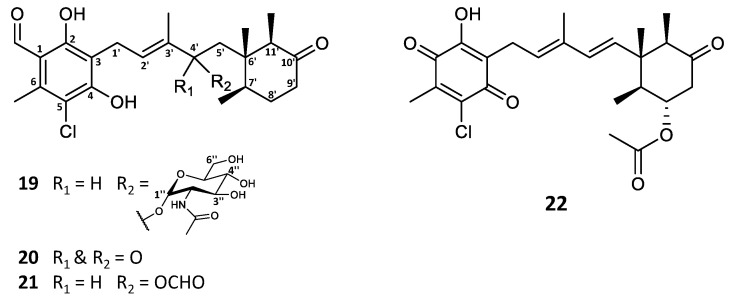
Structures of new ascochlorin analogues isolated from *Stilbella fimetaria*.

In the investigation of the secondary metabolite profile of *Stilbella fimetaria*, the fungus was initially cultivated on yeast extract sucrose (YES) and Czapek yeast extract agar (CYA) media for 9 days at 25 °C in the dark, resulting in a combined extract. The crude extract was fractionated by reversed phase (RP) flash chromatography, where three out of the six resulting fractions showed cytotoxic and antimicrobial activities [28]. The subsequent automated in-house MS/HRMS fungal secondary metabolite library search [29] revealed that along with the broad spectrum antimicrobials helvolic acid and ilicicolin H [28], smaller amounts of ascochlorin and related analogues could also be observed in the same bioactive fractions. In our previous study, different fungal cultivations were prepared to target different bioactive compound classes [28], and it was found that incubation on YES agar medium for 9 days at 25 °C in the dark results in higher and more diverse production of ascochlorin analogues. Analysis by UHPLC-DAD-QTOF-MS based on the characteristic UV absorption (λ_max_ 240, 290, and 346 nm) [2] and/or the similarity of the MS/HRMS fragmentation pattern to that of ascochlorin led to dereplication of the majority of the aforementioned analogues. Consequently, eight known compounds (**1, 3**, **5**, **6**, **8**–**11**) were isolated and their structures were confirmed by ^1^H NMR and HSQC data (Tables S1–S3). Moreover, four new ascochlorin analogues, ascochlorin *N*-acetylglucosamine (**19**), 4′-ketoascochlorin (**20**), and 4′,5′- dihydro-4′-formylascochlorin (**21**), as well as a novel benzoquinone equivalent of ilicicolin F (**3**), fimetarin A (**22**), were structure elucidated by 1D and 2D NMR spectroscopy (Figure 2 and Table 1).

### 2.1. Dereplication and Structural Elucidation of New Analogues

The molecular formula C_31_H_44_ClNO_10_ of ascochlorin *N*-acetylglucosamine (**19**) was established based on HRESIMS (*m*/*z* 626.2727 [M + H]^+^). The automated in-house MS/HRMS library search revealed that one of the major fragments of **19** matched against the data of an ascochlorin standard, suggesting that **19** contains the ascochlorin backbone, as well as an easily cleavable moiety with a molecular formula of C_8_H_15_NO_6_ (*m*/*z* 222.0975 [M + H]^+^). This hypothesis was further supported by the similarity of the UV absorption patterns of the two compounds. Subsequent ^1^H and ^13^C NMR data analysis confirmed the presence of the ascochlorin backbone with an additional amino sugar unit at the C-4′ position (Table 1).

**Figure 3:**
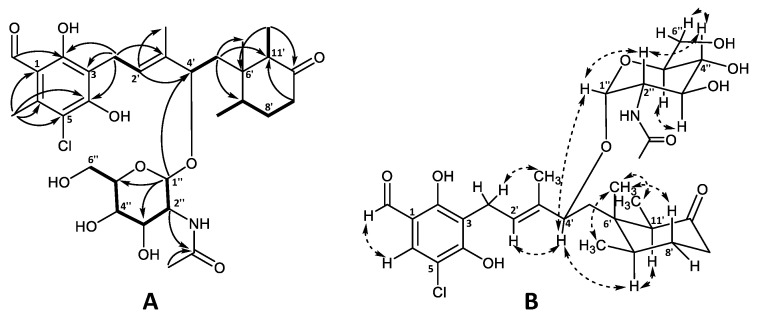
Key (**A**) COSY (bold), HMBC (solid arrows), and (**B**) NOESY (dashed arrows) correlations of ascochlorin *N*-acetylglucosamine (**19**).

The planar structure of the aglycone was confirmed by ^1^H and ^13^C chemical shift similarity to that of 4′5′-dihydro-4′-hydroxyascochlorin (**6**) characterized in this work (Table S3) as well as previously published values [1,6]. The relative stereochemistry of the cyclohexanone moiety was also found to be identical to that of ascochlorin (**1**) based on the NOESY correlations (Figure 3) [1].

The remaining six carbon atoms at δ_C_ 94.0, 55.3, 72.9, 72.6, 74.5, and 62.9 (C-1″–C-6″, respectively), as well as the amide carbonyl group at δ_C_ 173.6 (2″CO) and the methyl group at δ_C_ 22.8 (2″OCH_3_), were assigned to the amino sugar unit C_8_H_15_NO_6_. The corresponding methines at H-1″–H-5″ and a methylene at H-6″ constituted a single spin system, further supported by HMBC data (Figure 3A). The HMBC correlations from H-2″ (δ_H_ 3.87) and the singlet methyl group at 2″OCH_3_ (δ_H_ 2.00) to the amide carbonyl 2″CO (δ_C_ 173.6), indicated the NHCOCH_3_ functionality being attached at C-2″. Finally, the anomeric position of the amino sugar was assigned to the proton at H-1″ (δ_H_ 4.77) based on the strong HMBC correlation to C-4′ (δ_C_ 78.9) of the ascochlorin backbone, further supported by a strong correlation observed between H-4′ and H-1″ in the NOESY spectrum. The vicinal coupling constants of J_1″2″_ = 3.8 Hz, J_2″3″_ = 10.8 Hz, and J_3″4″_ and J_4″5″_ = 9.2 Hz indicated an equatorial position for H-1″ and axial positions for the remaining protons at H-2″ to H-5″ in a pyranose ring system. This was further supported with observed NOESY correlations between H-1″ and H-2″, H-2″ and H-4″, H-3″ and H-5″, as well as H-4″ and H-6″ in the NOESY spectrum (Figure 3B). Based on these observations, the amino sugar was identified as *N*-acetyl-α-D-glucosamine. This is only the second reported case of monosaccharide incorporation in the 4′5′-dihydro-4′-hydroxyascochlorin backbone, with the first being vertihemipterin A [1], both representing a rare fungal polyketide-sesquiterpenoid possessing a α-D-glucosamine unit.

**Figure 4:**
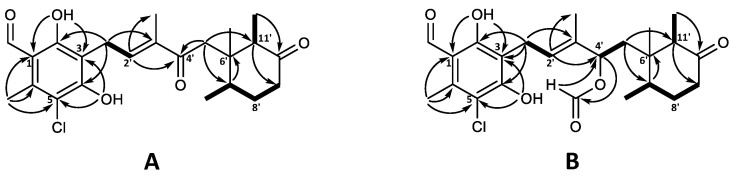
Key COSY (bold) and HMBC (solid arrows) correlations for (**A**) 4′-ketoascochlorin (**20**), and (**B**) 4′,5′-dehydro-4′-formylascochlorin (**21**).

The molecular formula of C_23_H_29_ClO_5_ for 4′-ketoascochlorin (**20**) was established based on HRESIMS (*m*/*z* 421.1776 [M + H]^+^). The observed UV absorption pattern was similar to that of ascochlorin, with λ_max_ 240, 290, and 340 nm. The ^1^H and ^13^C NMR data of **20** were highly comparable to the values of 4′5′-dihydro-4′-hydroxyascochlorin (**6**) obtained in this work (Table S3). The major difference was the change of C-4′ from δ_C_ 74.6 in **6** to the downfield value of δ_C_ 200.9 in **20**, indicating the presence of a keto group at this position. This is further supported by the significant downfield shift of nearby protons H-2′ (δ_H_ 6.62) and H-5′ (δ_H_ 2.74/2.51) for **20**, in comparison to that observed in **6** (δ_H_ 5.48 and δ_H_ 1.66/1.46, respectively). Correlations observed in the COSY and HMBC experiments further validated the expected backbone, as well as the keto group placement at C-4′ by key HMBC correlations from H-2′ (δ_H_ 6.62) and H-5′ (δ_H_ 2.74/2.51) (Figure 4A).

4′,5′-dihydro-4′-formylascochlorin (**21**) exhibited the expected UV absorption (λ_max_ 236, 292, and 342 nm) and a molecular formula of C_24_H_30_ClO_6_ established from HRESIMS (*m*/*z* 473.1699 [M + Na]^+^). The ^1^H and ^13^C NMR data of **21** were fairly identical to those of 4′5′-dihydro-4′-hydroxyascochlorin (**6**), with the main difference being an additional carbon atom (δ_C_ 160.2) and a proton (δ_H_ 8.03) observed, indicating the presence of a formyl ester (4′OCHO). A proton shift change at H-4′ from δ_H_ 4.21 in **6** to δ_H_ 5.49 in **21** suggested the formyl group to be placed at C-4′. Subsequent assignment of COSY and HMBC correlations confirmed the expected backbone and the formyl group placement at C4′ by HMBC correlations from the proton at 4′OCHO (δ_H_ 8.03) to C-4′ (δ_C_ 75.9) and from H-4′ (δ_H_ 5.49) to the carbonyl at 4′OCHO (δ_C_ 160.2; Figure 4B). However, we speculate that **21** might be an extraction artefact rather than a true biosynthetic product, due to the acidic extraction containing formic acid. Although no NOESY experiments for either **20** or **21** were acquired, the relative stereochemistry is expected to be the same as that of previously discussed analogues.

**Figure 5:**
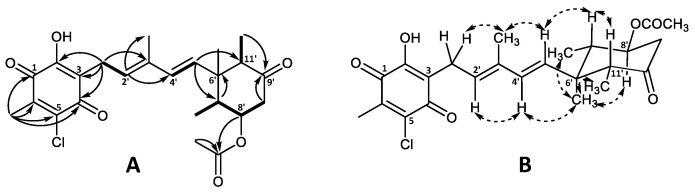
Key (**A**) COSY (bold), HMBC (solid arrows), and (**B**) NOESY (dashed arrows) correlations of fimetarin A (**22**).

The major halogen-containing ascochlorin analogue fimetarin A (**22**) displayed a different UV absorption pattern (λ_max_ 236 and 282 nm) to that of previously described for ascochlorin analogues (λ_max_ 240, 290, and 346 nm), and a molecular formula of C_24_H_29_ClO_6_ was established from HRESIMS (*m*/*z* 449.1725 [M + H]^+^). Notably, **22** showed an in-source fragment with corresponding molecular formula C_22_H_25_ClO_4_ (*m*/*z* 389.1518 [M + H]^+^), indicating the loss of an acetoxy moiety. The acquired spectroscopic data for **22** revealed similarities to ilicicolin F (**3**) data reported in this work (Table S1) as well as previously published data [8,30]. Namely, the ^1^H and ^13^C shifts of the sesquiterpene chain with an 8′-OAc moiety for **22** were comparable to those of **3** and were subsequently confirmed by COSY and HMBC correlations (Figure 5A). However, instead of the 5-chloroorcylaldehyde moiety observed in other ascochlorin analogues, the spectroscopic data of **22** revealed the presence of a 2-chloro-3-methyl-5-hydroxy-1,4-benzoquinone ring system. This was indicated by a downfield shift of the six remaining carbon atoms (Table 1), and HMBC correlations from H-1′ (δ_H_ 3.30) to C-2 (δ_C_ 154.9), C-3 (δ_C_ 120.6), and C-4 (δ_C_ 181.1), as well as correlations from 6′CH_3_ (δ_H_ 2.15) to C-1 (δ_C_ 182.6), C-5 (δ_C_ 139.9), C-6 (δ_C_ 142.5), and a weak ^4^*J* correlation to C-4 (Figure 5A).

The relative stereochemistry of **22** was proposed based on ^1^H coupling constants and NOESY correlations (Figure 5B). NOESY correlations of 3′CH_3_ (δ_H_ 1.89) with H-1′ (δ_H_ 3.30) and H-5′ (δ_H_ 5.44), as well as correlations between H-2′ (δ_H_ 5.45) and H-4′ (δ_H_ 5.95) indicated an E configuration for both double bonds at C-2′/C’3 and C’4/C’5. The coupling constant of J_7′8′_ = 11.5 Hz placed H-8′ and H-7′ in axial positions in the cyclohexanone ring. This was further supported by NOESY correlations between 6′CH_3_ (δ_H_ 0.74) with 7′CH_3_ (δ_H_ 0.87), H-8′ (δ_H_ 4.84), 11′CH_3_ (δ_H_ 0.82) as well as H-4′, placing all three methyl groups on the same side of the plane, opposite to H-7′ (δ_H_ 2.09), H-11′ (δ_H_ 2.62), and 8′OAc. The latter was further supported by NOESY correlations of H-7′ with H-5′and H-11′.

### 2.2. UV-VIS- and MS-Based Dereplication of Fimetarin A Analogues

**Figure 6:**
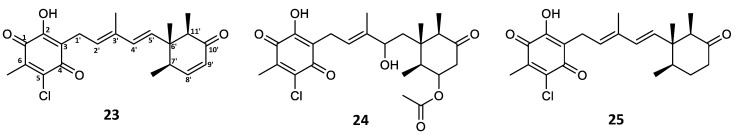
Fimetarin B (**23**), C (**24**), and D (**25**) tentative structures proposed based on MS/HRMS.

Expanding the investigation into the production of other potential fimetarin A (**22**) analogues, the crude extract along with initial fractions were screened for candidates exhibiting the same UV absorption pattern as **22** (λ_max_ 236 and 282 nm), molecular formulae containing an even number of carbon atoms (in contrast to the odd number observed in ascochlorin analogues), and a single chlorine atom. In addition to **22**, three other compounds met the selected criteria with the assigned molecular formulae, namely **23** with C_22_H_25_ClO_4_ (*m*/*z* 389.1515 [M + H]^+^), **24** with C_24_H_31_ClO_7_ (*m*/*z* 467.1831 [M + H]^+^), and **25** with C_22_H_27_ClO_4_ (*m*/*z* 391.1673 [M + H]^+^). Based on the comparison of MS/HRMS spectra of these dereplicated compounds with those of **22**, the structures of three new fimetarin A analogues could be tentatively proposed, namely fimetarins B (**23**), C (**24**), and D (**25**) (Figure 6). We hypothesize that their expected stereochemistry is the same as that of all previously discussed ascochlorin analogues.

**Figure 7:**
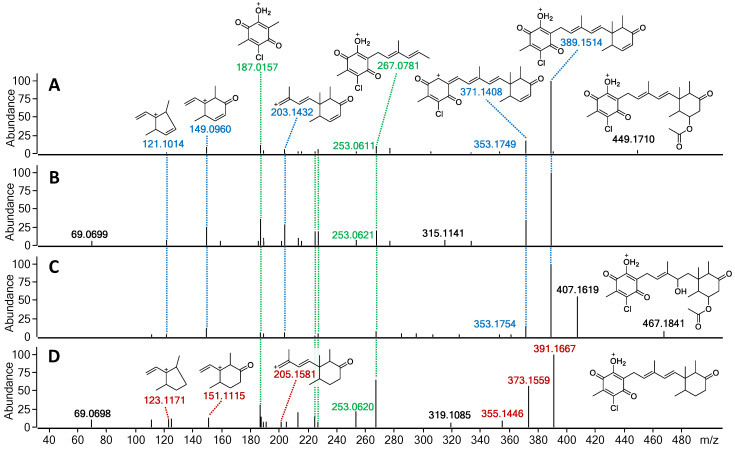
MS/HRMS spectra at 10eV with ion abundance threshold of 2% for (**A**) fimetarin A (**22**), (**B**) fimetarin B (**23**), (**C**) fimetarin C (**24**), and (**D**) fimetarin D (**25**). Highlighted in green are the fragment ions common in all four compounds, in blue-fragment ions shared among **22**–**24**, and in red-fragment ions unique to **25**.

The fragmentation patterns of **23** and **24** were found to be nearly identical to that of **22** (highlighted in blue and green; Figure 7 and Figure S8). The initial acetoxy loss in both **22** and **24**, and an additional water moiety loss for **24** results in both compounds sharing the same major daughter ion of *m*/*z* 389.1514, whereas the same *m*/*z* ratio corresponds to the parent ion in **23**. Subsequent sesquiterpene chain fragmentation in all three compounds occurs in the same manner, only with small discrepancies in fragment intensity and fragmentation corresponding to initial terpene ring opening. This suggests **23** to be the deacetoxylated analogue of **22** and benzoquinone equivalent to ilicicolin E (**9**). For **24**, the proposed structure is the hydrated analogue of **22**, with an alcohol group at C-4′. Since no ascochlorin analogues have been previously reported to be simultaneously substituted at both C-4′ and C-8′, we speculate that **24** is most likely yet another acidic extraction artefact. In contrast to **23** and **24**, the MS/HRMS of **25** showed only partial similarity to **22,** with the observed fragment ions being either identical (highlighted in green) or 2 Da higher (highlighted in red), indicating one degree of unsaturation less compared to **23** (Figure 7). Upon analysis of the individual MS/HRMS fragmentation patterns, it was found that all the fragments corresponding to the benzoquinone moiety with an associated linear terpenoid chain (highlighted in green) were common among all four compounds (**22**–**25**); however, the fragments corresponding to the cyclized terpene part of the molecule, irrespective of the associated linear terpene side chain, were always 2 Da higher for **25** (highlighted in red) in contrast to the other three compounds **22**–**24** (highlighted in blue; Figure 7 and Figure S8). This indicated the loss of a degree of unsaturation within the terpene ring, most likely at the C-8′/C-9′ positions, resulting in a benzoquinone equivalent of ilicicolin C (**5**).

The crude extract along with initial fractions were additionally screened for the presence of 3-methyl-5-hydroxy-1,4-benzoquinone, as a potential starter unit for fimetarin biosynthesis, as well as the initial pathway intermediate with a C-3 substituted sesquiterpene side chain, however with unsuccessful results. This suggests the possibility of unknown enzymatic activity that is either modifying the final pathway products or intercepting the biosynthetic pathway of ascochlorin at the early stages of biosynthesis. In case of the latter, the potential reaction mechanism might be similar to that observed in oosporein biosynthesis, where salicylate hydroxylase (OpS4) activity results in orsellinic acid conversion to a substituted 1,4-benzoquinone moiety via an oxidative decarboxylation [31].

### 2.3. Biological Structure-Activity Relationship of Ascochlorin Analogues

Compounds **1**, **3**, **5**, **6**, **8**–**11**, and **19**–**22** were tested for their antifungal and antibacterial activity against *Aspergillus fumigatus*, *Candida albicans*, methicillin resistant *Staphylococcus aureus* (MRSA), and *Escherichia coli* (Table 2). Strong inhibitory activities (1.25–4.1 μg/mL) were observed for compounds **1**, **3**, and **9**, and moderate activity (20 μg/mL) for **22** was observed against *A. fumigatus*. Comparison of the structural activity relationship of **1**, **3**, and **9** suggested that the 5-chloroorcylaldehyde moiety along with the two double bonds at C-2′/C-3′ and C-4′/C-5′ with an E configuration are essential for the bioactivity against *A. fumigatus*, since no activities were observed in any of the other tested analogues with a saturated C-4′/C-5′ bond. Moreover, slight modifications of the ascochlorin (**1**) backbone at the cyclic terpene part, namely acetylation at C-8′ or dehydrogenation at C-8′/C-9′, did not significantly affect compound activity. This is further supported by the significantly increased MIC value for **22**, with the only difference to **3** being the benzoquinone moiety. The same four compounds (**1**, **3**, **9**, and **22**) were also active against *C. albicans*, where **3** exhibited moderate activity (6.6–13.3 μg/mL), whereas the rest (**1**, **9**, and **22**) showed weak activities (66.6–133.3 μg/mL). Here, the same double bond positioning at C-2′/C-3′ and C-4′/C-5′ along with acetylation at C-8′ are proposed to be essential for the observed bioactivity, since both non-acetylated analogues **1** and **9**, showed weaker MIC values. Interestingly, **22** is in the same bioactivity range as **1** and **9,** leading to speculations that the presence of the acetyl group at C-8′ is equally if not more important than the presence of a 5-chloroorcylaldehyde moiety for the activity against *C. albicans.* In an anti-MRSA assay, **5** exhibited the strongest (1.25–2.5 μg/mL), whereas **8** and **11** showed moderate (26.6 and 40 μg/mL, respectively) and **20** exhibited weak (93.3 μg/mL) antibacterial activities. This suggests that the presence of non-modified single bonds at C-4′/C-5′ and C-8′/C-9′ is essential, since none of the other unsaturated analogues and/or modifications at either or both of these positions showed bioactivity at the tested concentrations. Moreover, with **8** being the non-halogenated precursor of **5**, it is proposed that the presence of a chlorine atom is preferred for the higher potency of the compounds against *S. aureus* (MRSA). Finally, the more than 3-fold increase in MIC values from **11** to **10**, with a single difference of an oxidized keto group, suggests that the keto group at C-10′ is essential for ascofuranone (**11**) bioactivity.

## 3. Materials and Methods

### 3.1. General Experimental Procedures

All samples were analyzed on an Agilent Infinity 1290 UHPLC system (Agilent Technologies, Santa Clara, CA, USA) equipped with a diode array detector (DAD), monitoring between 190 and 640 nm. Separation was achieved on an Agilent Poroshell 120 phenyl-hexyl column (150 × 2.1 mm, 1.9 μm particles) with a flow rate of 0.35 mL/min at 40 °C, using a linear acetonitrile (MeCN)/water (both buffered with 20 mM FA) gradient of 10% to 100% MeCN in 10min, followed by 2min flush at 100% MeCN, returning to starting conditions in 0.1 min, and equilibration at 10% for 2 min before the following run. It was coupled to an Agilent 6545 QTOF MS equipped with a Dual Jet Stream ESI source with the drying gas temperature of 250 °C and gas flow of 8 L/min and sheath gas temperature of 300 °C and flow of 12 L/min, capillary voltage 4000 V, and nozzle voltage of 500 V. The mass spectrometer was operated in positive polarity, recording centroid data in the *m*/*z* range 100 to 1700 for MS mode, and 30–1700 for MS/MS mode, with an acquisition rate of 10 spectra/s. Automated MS/HRMS was performed for ions detected in the full scan above 50,000 counts, with a cycle time of 0.5 s and quadrupole width of *m*/*z* ±0.65 using fixed CID energies of 10, 20, and 40 eV with a maximum of three precursor ions per cycle. A lock mass solution of 70% MeOH was infused in the second ESI sprayer, with an extra LC pump at a flow of 15 μL/min using a 1:100 splitter. The solution contained 1 μM tributylamine (Sigma-Aldrich, St. Louis, MO, USA) and 10 μM hexakis(2,2,3,3-tetrafluoropropoxy)phosphazene (Apollo Scientific Ltd., Cheshire, UK) as lock masses. The [M + H]^+^ ions of both compounds (*m*/*z* 186.2216 and 922.0098, respectively) were used.

1D and 2D NMR spectra were acquired on a Bruker Avance 800 MHz spectrometer (Bruker, Billerica, MA, USA), using standard sequence pulses. Samples were analyzed in a 3 mm TCl cryoprobe using either deuterated methanol (CD_3_OD) or chloroform (CDCl_3_), and referred to the residual solvent signals δ_H_ = 7.26 ppm and δ_C_ = 77.16 ppm for CDCl_3_, and δ_H_ = 3.31 ppm and δ_C_ = 49.0 ppm for CD_3_OD. J-couplings are reported in hertz (Hz) and chemical shifts (δ) in ppm.

Optical rotations were measured in methanol (MeOH) on a PerkinElmer 341 Polarimeter (PerkinElmer, Waltham, MA, USA).

### 3.2. Fungal Strain and Cultivation

*Stilbella fimetaria* (IBT 28361) is a marine-derived fungus from the IBT culture collection at DTU Bioengineering. For large-scale cultivation, the fungus was cultivated with 3-point inoculation on 200 YES agar plates, and incubated for 9 days at 25 °C in the dark.

### 3.3. Extraction and Isolation

For the large-scale extraction, the agar plates were extracted twice with acidic (1% FA) EtOAc. Liquid-liquid partition was then performed on the dried crude extract with 90% MeOH:water and heptane, resulting in two phases; the 90% MeOH:water fraction was then diluted with water up to 50% and further extracted with dichloromethane (DCM), resulting in three phases overall. The DCM phase was dried before loading into a 50 g SNAP column (Biotage, Uppsala, Sweden) with diol material (Isolute diol, Biotage). Crude fractionation was performed using an Isolera One automated flash system (Biotage) with stepwise increments of 25% and 50% at 40 mL/min in a heptane-DCM-EtOAc-MeOH system, starting at 100% heptane and finishing at 100% MeOH, resulting in 10 fractions overall of 300 mL each. Selected fractions of interest were further fractionated with a Waters 600 Controller (Milford, MA, USA) coupled to a Waters 996 Photodiode Array Detector, with UV monitoring at 230 and 280 nm; a linear gradient of 60% to 80% MeOH/water (both +50 ppm TFA) over 20 min at a flow rate of 4 mL/min was run on a Kinetex RP C18 column (5 μm, 100 Å, 250 × 10 mm, Phenomenex) to yield **1**, **5**, and **11**. Additional separation for the remaining compounds was achieved on an Agilent Infinity 1290 HPLC-DAD (Agilent Technologies, Santa Clara, CA, USA) system, with UV monitoring at 230 and 280 nm; a linear gradient of 45% to 65% MeOH/water (both +50 ppm TFA) over 30 min at a flow rate of 4 mL/min and a column temperature at 40 °C using a Kinetex RP C18 column (5 μm, 100 Å, 250 × 10 mm, Phenomenex).

*Ascochlorin* (**1**): pale yellow powder; UV (MeCN) λ_max_ 240, 292, 342 nm; ^1^H and ^13^C NMR data, see Table S1; HRESIMS *m*/*z* 405.1826 [M + H]^+^ (calculated for C_23_H_29_ClO_4_, *m*/*z* 405.1827).

*Ilicicolin F* (**3**): white powder; UV (MeCN) λ_max_ 240, 292, 341 nm; ^1^H and ^13^C NMR data, see Table S1; HRESIMS *m*/*z* 463.1882 [M + H]^+^ (calculated for C_25_H_31_ClO_6_, *m*/*z* 463.1882).

*Ilicicolin C* (**5**): white powder; UV (MeCN) λ_max_ 239, 293, 339 nm; ^1^H and ^13^C NMR data, see Table S1; HRESIMS *m*/*z* 407.1983 [M + H]^+^ (calculated for C_23_H_31_ClO_4_, *m*/*z* 407.1984).

*4′,5′-dehydro-4′-hydroxyascochlorin* (**6**): pale yellow powder/oil; [α]${}_{D}^{20}$ +36.6° (c 0.8, MeOH); UV (MeCN) λ_max_ 235, 293, 341 nm; ^1^H and ^13^C NMR data, see Table S3; HRESIMS *m*/*z* 445.1752 [M + Na]^+^ (calculated for C_23_H_31_ClO_5_Na, *m*/*z* 445.1752).

*LL-Z1272ε* (**8**): pale yellow powder; UV (MeCN) λ_max_ 234, 297, 335 nm; ^1^H and ^13^C NMR data, see Table S1; HRESIMS *m*/*z* 373.2376 [M + H]^+^ (calculated for C_23_H_32_O_4_, *m*/*z* 373.2373).

*Ilicicolin E* (**9**): white powder; UV (MeCN) λ_max_ 242, 291, 342 nm; ^1^H and ^13^C NMR data, see Table S1; HRESIMS *m*/*z* 403.1672 [M + H]^+^ (calculated for C_23_H_27_ClO_4_, *m*/*z* 403.1671).

*Ascofuranol* (**10**): yellow oil; [α]${}_{D}^{20}$ −5° (c 0.08, MeOH); UV (MeCN) λ_max_ 238, 293, 340 nm; ^1^H and ^13^C NMR data, see Table S2; HRESIMS *m*/*z* 423.1934 [M + H]^+^ (calculated for C_23_H_31_ClO_5_, *m*/*z* 423.1933).

*Ascofuranone* (**11**): yellow oil; UV (MeCN) λ_max_ 241, 292, 344 nm; ^1^H and ^13^C NMR data, see Table S2; HRESIMS *m*/*z* 421.1778 [M + H]^+^ (calculated for C_23_H_29_ClO_5_, *m*/*z* 421.1776).

*Ascochlorin N-acetylglucosamine* (**19**): pale yellow solid; UV (MeCN) λ_max_ 230, 292, 344 nm; ^1^H and ^13^C NMR data, see Table 1; HRESIMS *m*/*z* 626.2727 [M + H]^+^ (calculated for C_31_H_44_ClNO_10_, *m*/*z* 626.2727); MS/HRMS data, see Figure S1.

*4′-ketoascochlorin* (**20**): yellow solid; [α]${}_{D}^{20}$ 0° (c 0.07, MeOH); UV (MeCN) λ_max_ 240, 290, 340 nm; ^1^H and ^13^C NMR data, see Table 1; HRESIMS *m*/*z* 421.1776 [M + H]^+^ (calculated for C_23_H_29_ClO_5_, *m*/*z* 421.1776); MS/HRMS data, see Figure S2.

*4′,5′-dihydro-4′-formylascochlorin* (**21**): pale yellow oil; [α]${}_{D}^{20}$ +30° (c 0.17, MeOH); UV (MeCN) λ_max_ 236, 292, 342 nm; ^1^H and ^13^C NMR data, see Table 1; HRESIMS *m*/*z* 473.1699 [M + Na]^+^ (calculated for C_24_H_30_ClO_6_Na, *m*/*z* 473.1701); MS/HRMS data, see Figure S3.

*Fimetarin A* (**22**): yellow solid; [α]${}_{D}^{20}$ −15° (c 0.05, MeOH); UV (MeCN) λ_max_ 236, 282 nm; ^1^H and ^13^C NMR data, see Table 1; HRESIMS *m*/*z* 449.1725 [M + H]^+^ (calculated for C_24_H_29_ClO_6_, *m*/*z* 449.1725); MS and MS/HRMS data, see Figure S4.

### 3.4. Antibacterial and Antifungal Assays

Compounds **1**–**3**, **5**, **8**–**11**, and **19**–**22** were evaluated for their antimicrobial activity against the growth of fungi (*A. fumigatus* ATCC46645), yeast (*C. albicans* ATCC64124), Gram-positive (methicillin-resistant *S. aureus* (MRSA) MB5393), and Gram-negative (*E. coli* ATCC 25922) bacteria, based on previously described methods [32,33,34]. Briefly, each compound was serially diluted in DMSO with a dilution factor of 2 to provide 10 concentrations with varying starting concentrations depending on individual sample availability (53.3–186.7 μg/mL, see Table 2). The MIC was defined as the lowest concentration of tested compound that inhibited ≥90% of the growth of a microorganism after overnight incubation. All experiments were performed in triplicates. The Genedata Screener software (Genedata, Inc., Basel, Switzerland) was used to process and analyze the data and to calculate the RZ’ factor, which predicts the robustness of an assay [35]. In all experiments performed in this work, the RZ’ factor obtained was between 0.87 and 0.98.

## 4. Conclusions

In summary, a marine-derived isolate of *Stilbella fimetaria* was found to be a prolific producer of a series of novel bioactive ascochlorin family compounds. Notably, a new sub-class of compounds, namely fimetarins A–D (**22**–**25**), comprised of a sesquiterpene-substituted seven carbon 1,4-benzoquinone moiety, was described for the first time. This implies that an additional oxidative decarboxylation step must be involved in the early stages of fimetarin biosynthesis, as compared to ascochlorins with an eight carbon 5-chloroorcylaldehyde moiety.

Upon antimicrobial activity testing, it was found that fimetarin A (**22**) expressed lower inhibitory activity in comparison to its orcylaldehyde equivalent, ilicicolin F (**3**), clearly indicating the orcylaldehyde moiety to be part of the pharmacophore. Additionally, it was proposed that the presence of two conjugated double bonds at C-2′/C-3′ and C-4′/C-5′ is essential for strong antifungal activity against *A. fumigatus* and *C. albicans*, whereas the single, untailored bonds at C-4′/C-5′ and C-8′/C-9′ are important for the antibacterial activity against *S. aureus* MRSA.

## Figures and Tables

**Table 1:** ^1^H and ^13^C NMR data for ascochlorin *N*-acetylglucosamine (**19**), 4′-ketoascochlorin (**20**), 4′,5′-dihydro-4′-formylascochlorin (**21**), and fimetarin A (**22**).

Pos.	19 ^a^		20 ^b^		21 ^b^		22 ^a^	
	δ_C_	δ_H_, mult (J)	δ_C_	δ_H_, mult (J)	δ_C_	δ_H_, mult (J)	δ_C_	δ_H_, mult (J)
**1**	112.9		113.7		112.9		182.6	
**1CHO**	193.5	9.90, s	193.2	10.17, s	193.2	10.12, s		
**2**	163.3		162.2		162.0		154.9	
**2OH**				12.76, s		12.67, s		
**3**	114.4		111.7		112.9		120.6	
**4**	163.3		156.1		160.2		181.1	
**4OH**				6.44, brs		6.38, brs		
**5**	117.2		113.1		112.9		139.9	
**6**	139.6		138.5		137.9		142.5	
**6CH_3_**	14.8	2.51, s	14.5	2.63, s	14.4	2.59, s	13.3	2.15, s
**1′a**	22.7	3.53, dd(13.6, 9.0)	23.0	3.62, d(7.1)	21.5	3.41, q(7.9)	23.5	3.30, d(7.6)
**1′b**	22.7	3.34, dd(13.6, 9.0)			21.5	3.39, q(7.0)		
**2′**	130.8	5.70, t(7.3)	137.9	6.62, t(7.2)	125.9	5.61, t (7.4)	128.6	5.45, m
**3′**	134.7	-	138.0		134.7		135.5	
**3′CH_3_**	11.2	1.74, s	11.3	1.93, s	11.6	1.82, s	13.0	1.89, s
**4′**	78.9	4.31, dd(8.4, 4.2)	200.9		75.9	5.49, dd(7.0, 4.6)	135.4	5.95, d(16.0)
**4′OCHO**					160.2	8.03, s		
**5′a**	40.5	1.74, m	41.3	2.74, d(17.8)	39.1	1.83, dd(15.8, 7.2)	136.0	5.44, d(16.0)
**5′b**	40.5	1.60, dd(15.6, 4.2)	41.3	2.51, d(17.8)	39.1	1.59, dd(15.8, 4.5)		
**6′**	44.9		44.1		43.8		46.8	
**6′CH_3_**	16.5	0.49, s	15.5	0.59, s	15.4	0.55, s	11.9	0.74 s
**7′**	37.2	2.14, m	35.4	2.59, m	36.4	1.95, m	46.2	2.09, dq(11.5, 6.7)
**7′CH_3_**	16.2	0.96, d(6.6)	15.6	0.81, d (6.8)	15.4	0.95, d(6.7)	12.8	0.87, d(6.7)
**8′a**	32.2	1.45, dq(13.1, 4.9)	30.7	1.80, m	30.9	1.79, m	75.2	4.84, m
**8′b**	32.2	1.75, m	30.7	1.56, qd(13.4, 5.2)	30.9	1.54, qd(13.7, 4.8)		
**8′CO**							172.2	
**8′COCH_3_**							21.1	2.05, s
**9′a**	42.6	2.02, ddd(13.4, 4.9, 2.1)	41.5	2.42, td(13.5, 7.3)	41.3	2.26, ddd(13.6, 4.8, 1.9)	48.1	2.74, dd(13.2, 5.6)
**9′b**	42.6	1.96, dt(13.4, 6.8)	41.5	2.32, ddd(13.6, 4.8, 1.5)	41.3	2.16, tdd(13.7, 7.1, 1.0)		2.58, m
**10′**	216.7		216.1		216.7		210.4	
**11′**	51.5	2.49, q(6.6)	50.1	3.32, q(6.7)	50.3	2.51, q(6.7)	54.6	2.62, m
**11′CH_3_**	8.9	0.67, d(6.6)	8.1	0.86, d(6.5)	7.9	0.79, d(6.7)	9.3	0.82, d(6.7)
**1″**	94.0	4.77, d(3.8)						
**2″**	55.3	3.87, dd(10.8,3.8)						
**2″CO**	173.6							
**2″CH_3_**	22.8	2.00, s						
**3″**	72.9	3.68, m						
**4″**	72.6	3.34, t(9.2)						
**5″**	74.5	3.66, m						
**6″a**	62.9	3.80, dd(11.4, 1.8)						
**6″b**	62.9	3.69, m						

^a^ CD_3_OD; ^b^ CDCl_3_.

**Table 2:** Antifungal and antibacterial activities of compounds **1, 3**, **5**, **6**, **8**–**11**, and **19**–**22**.

Compound	MIC (μg/mL (μM))			
	*A. fumigatus*	*C. albicans*	*MRSA*	*E. coli*
**1**	1.25–2.5	80	>80	>80
**3**	1.66–3.33	6.66–13.33	>53.33	>53.33
**5**	>160	>160	1.25–2.5	>160
**6**	>128	>128	>128	>128
**8**	>106.70	>106.70	26.66	>106.70
**9**	4.1	66.67–133.3	>133.3	>133.3
**10**	>133.33	>133.33	133.3	>133.3
**11**	>160	>160	40	>160
**19**	>128	>128	>128	>128
**20**	>186.70	>186.70	93.33	>186.70
**21**	>160	>160	160	>160
**22**	20	80	>160	>160

## Data Availability

Data is contained within the article and Supplementary Material.

## Supplementary Materials

The following are available online at https://www.mdpi.com/1660-3397/19/2/46/s1, The supplementary material containing 1D and 2D NMR spectra for all isolated compounds as well as MS/HRMS spectra for new compounds.
