# Supplementary material for: Bioactive Ascochlorin Analogues from the Marine-Derived Fungus Stilbella fimetaria
Source: Mar Drugs. 2021 Jan 20;19(2):46. doi: 10.3390/md19020046 (PMC7909580; doi:10.3390/md19020046)
Supplement: Supplementary file 1 [file marinedrugs-19-00046-s001.pdf]

Supplementary Information

Bioactive Ascochlorin Analogues from the Marine-Derived Fungus *Stilbella fimetaria*

Karolina Subko [1], Sara Kildgaard [2], Francisca Vicente [3], Fernando Reyes [3], Olga Genilloud [3], and Thomas O. Larsen [1,*]

[1] DTU Bioengineering, Technical University of Denmark, Søltofts Plads 221, DK-2800 Kgs. Lyngby, Denmark; karosu@dtu.dk (K.S.);
[2] Department of Biology, University of Copenhagen, Universitetsparken 15, DK-2100 København Ø, Denmark; sara.kildgaard@bio.ku.dk (S.K.);
[3] Fundación MEDINA, Avda. del Conocimiento, 34, 18016 Armilla, Granada, Spain; francisca.vicente@medinaandalucia.es (F.V.); fernando.reyes@medinaandalucia.es (F.R.); olga.genilloud@medinaandalucia.es
* Correspondence: tol@bio.dtu.dk; Tel.: +45-4525-2632 (T.O.L)

Table of contents

Table S1. ^{1}H and ^{13}C NMR data for known ascochlorin analogues (**1**, **3**, **5**, **8-9**).
Table S2. ^{1}H and ^{13}C NMR data for ascofuranol (**10**) and ascofuranone (**11**).
Table S3. ^{1}H and ^{13}C NMR data for 4’5’-dihydro-4’-hydroxyascochlorin (**6**) in CD_3OD and $CDCl_3$.
Figure S1. MS/HRMS spectra for ascochlorin N-acetylglucosamine (**19**).
Figure S2. MS/HRMS spectra for 4’-ketoascochlorin (**20**).
Figure S3. MS/HRMS spectra for 4',5'- dihydro-4'-formylascochlorin (**21**).
Figure S4. MS/HRMS spectra for fimetarin A (**22**).
Figure S5. MS/HRMS spectra for fimetarin B (**23**).
Figure S6. MS/HRMS spectra for fimetarin C (**24**).
Figure S7. MS/HRMS spectra for fimetarin D (**25**).
Figure S8. Proposed fragmentation patterns for fimetarins A-D (**22-25**).
Figure S9. ^{1}H and HSQC spectra for ilicicolin D (**1**) in $CDCl_3$.
Figure S10. ^{1}H and HSQC spectra for ilicicolin F (**3**) in $CDCl_3$.
Figure S11. ^{1}H and HSQC spectra for ilicicolin C (**5**) in $CDCl_3$.
Figure S12. 1D and 2D NMR spectra for 4’5’-dihydro-4’-hydroxyascochlorin (**6**) in CD_3OD.
Figure S13. 1D and 2D NMR spectra for 4’5’-dihydro-4’-hydroxyascochlorin (**6**) in $CDCl_3$.
Figure S14. ^{1}H and HSQC spectra for LL-Z1272ε (**8**) in $CDCl_3$.
Figure S15. ^{1}H and HSQC spectra for ilicicolin E (**9**) in $CDCl_3$.
Figure S16. ^{1}H and HSQC spectra for ascofuranol (**10**) in $CDCl_3$.
Figure S17. ^{1}H and HSQC spectra for ascofuranone (**11**) in $CDCl_3$.
Figure S18. 1D and 2D NMR spectra for ascochlorin N-acetylglucosamine (**19**) in CD_3OD.
Figure S19. 1D and 2D NMR spectra for 4’-ketoascochlorin (**20**) in $CDCl_3$.
Figure S20. 1D and 2D NMR spectra for 4',5'- dehydro-4'-formylascochlorin (**21**) in $CDCl_3$.
Figure S21. 1D and 2D NMR spectra for fimetarin A (**22**) in CD_3OD.

Table S1. ^{1}H and ^{13}C NMR data for known ascochlorin analogues (**1**, **3**, **5**, **8-9**).

	Ascochlorin **(1)**		Ilicicolin F **(3)**		Ilicicolin C **(5)**		LL-Z1272Ɛ **(8)**		Ilicicolin E **(9)**	
**Pos.**	δC	δH, mult. (J in Hz)	δC	δH, mult. (J in Hz)	δC	δH, mult. (J in Hz)	δC	δH, mult. (J in Hz)	δC	δH, mult. (J in Hz)
**1CHO**		10.14, s		10.15, s		10.14, s		10.08, s		10.14, s
**2OH**		12.70, s		12.71, s		12.69, s		12.74, s		12.70, s
**4OH**		6.42, s		6.38, s		6.43, s		6.15, br s		6.40, s
**5**							110.6	6.21, s		
**6CH₃**	14.4	2.60, s	14.4	2.61, s	14.4	2.60, s	18.0	2.50, s	14.4	2.60,s
**1'**	22.1	3.53, d (7.4)	22.2	3.54, d (7.5)	22.0	3.39, d (7.2)	21.2	3.39, d (7.2)	22.1	3.54, d (7.5)
**2'**	127.4	5.52, t (7.5)	128.2	5.55, t (7.4)	120.8	5.24, t (5.2)	121.1	5.28, t (7.2)	128.0	5.54, t (7.4)
**3'CH₃**	12.5	1.92, s	12.6	1.92, s	16.3	1.80, s	16.5	1.84, s	12.5	1.93, s
**4'a**	133.1	5.89, d (16.0)	134.1	5.92, d (16.0)	32.5	1.98, m	32.6	2.05, td(13.2, 4.8)	128.1	5.98,dd(10.1, 3.2)
**4'b**					32.5	1.84, m	32.6	1.90, td(13.1, 4.6)		
**5'a**	135.6	5.37, d (16.0)	134.1	5.32, d (16.0)	35.5	1.42, qd(14.6, 4.6)	35.6	1.44, dd(14.4, 4.5)	134.3	5.42, d(16.0)
**5'b**					35.5	1.35, qd(14.5, 4.4)	35.6	1.38, m		
**6'CH₃**	10.3	0.69, s	11.3	0.73, s	15.3	0.56, s	15.2	0.57, s	9.9	0.79, s
**7'**	40.7	1.93, m	45.3	1.99, m	36.0	1.97, m	36.1	1.98, m	41.9	2.63, m
**7'CH₃**	16.2	0.80, d (6.6)	12.4	0.86, d (6.8)	15.0	0.87, d (6.8)	15.1	0.88, d (6.7)	15.1	0.98, d (7.5)
**8'a**	31.0	1.92, m	73.6	4.89, td (11.1,5.6)	30.9	1.83, m	30.8	1.85, m	152.1	6.55, dd(10.1,2.0)
**8'b**	31.0	1.61, qd (13.8, 4.9)			30.9	1.60, m	30.8	1.62, m		
**8'OCH₃**			21	2.06, s						
**9'a**	41.5	2.43, ddd(13.7,6.9,1.0)	47.1	2.87, dd(13.3,5.7)	41.5	2.31, m	41.5	2.33, m	134.1	5.98, d (15.8)
**9'b**	41.5	2.36, ddd(13.6,5.1,1.8)	47.1	2.42, m						
**11'**	53.5	2.40, m	53.8	2.41, m	50.4	2.45, q (6.7)	50.4	2.46, q (6.7)	51.8	2.45, q (6.7)
**11'CH₃**	8.8	0.83, d (6.7)	8.8	0.86, d (6.8)	7.5	0.90, d (6.7)	7.5	0.91, d (6.8)	50.4	2.46, q (6.7)

Table S2. ^1H and ^{13}C NMR data for ascofuranol (**10**) and ascofuranone (**11**).

	Ascofuranol (**10**)		Ascofuranone (**11**).	
**Pos.**	δC	δH, mult. (J in Hz)	δC	δH, mult. (J in Hz)
**1CHO**	10.13, s			10.11, s
**2OH**	12.69, s			12.66, s
**4OH**	7.01, brs			6.56, brs
**$6CH_3$**	14.3	2.59, s	14.3	2.58, s
**1'**			21.9	3.37, d (7.1)
**2'**	121.5	5.15, t (6.9)	121.2	5.19, td (7.2, 1.2)
**$3'CH_3$**	15.9	1.76, s	16.0	1.77, s
**4'a**	39.1	2.05, t (7.4)	38.8	2.02, m
**5'a**	25.5	2.17, m	25.8	2.14, m
**5'b**	25.5	2.10, m		
**6'**	124.5	5.49, t (7.2)	128.4	5.49, t (7.1)
**$7'CH_3$**	12.3	1.59, s	11.1	1.61,s
**8'a**	79.7	4.30, t (7.2)	77.7	4.50, dd (10.3, 6.1)
**9'a**	39.0	2.39, m	39.8	2.40, dd (18.2, 16.2)
**9'b**	39.0	1.78, m	39.8	2.34, dd (18.2, 10.3)
**10'**	78.4	3.94, dd (6.1, 3.9)		
**11'**	21.8	3.37, qd (15.0, 7.2)		
**$11'CH_3a$**	22.3	1.28, s	21.8	1.20, s
**$11'CH_3b$**	25.9	1.20, s	24.1	1.26, s

Table S3. ^{1}H and ^{13}C NMR data for 4'5'-dihydro-4'-hydroxyascochlorin (**6**) in CD_3OD and $CDCl_3$.

	**6** in CD_3OD		**6** in $CDCl_3$	
**Pos.**	δC	δH, mult. (J in Hz)	δC	δH, mult. (J in Hz)
**1**	114.6	-	113.5	
**1CHO**	195.6	10.12 s		10.11, s
**2**	162.8	-	162.0	
**2OH**				12.67, s
**3**	115.1	-	113.5	
**4**	159.4	-	156.0	
**4OH**				6.50, brs
**5**	115	-	113.5	
**6**	140.1	-	137.8	
**6CH₃**	14.7	2.57 s	14.4	2.58, s
**1'a**	22.5	3.54 dd(13.8,8.9)	21.4	3.39, d (7.4)
**1'b**		3.29 m		
**2'**	125	5.60 t(7.3)	122.8	5.48, t (7.1)
**3'**	140.3	-	139.1	
**3'CH₃**	11.4	1.83 s	11.3	1.81, s
**4'**	75.4	4.23 dd(7.2,5.1)	74.6	4.21, dd (7.4, 4.2)
**4'CHO**				
**5'a**	42.1	1.59 dd(15.6,7.2)	41.2	1.66, dd (15.4, 7.3)
**5'b**		1.52 dd(15.6, 5.1)	41.2	1.46, dd (15.4, 4.2)
**6'**	45	-	44.1	
**6'CH₃**	16.4	0.49 s	15.5	0.54, s
**7'**	37.5	2.20 m	36.6	2.30, m
**7'CH₃**	16	0.96 d(6.6)	15.7	0.96, d (6.7)
**8'a**	32.2	1.48 dq(13.1,4.9)	31.1	1.80, m
**8'b**		1.75 m	31.1	1.54, qd (13.3, 5.1)
**9'a**	42.6	2.07 m	41.5	2.27, ddd (13.8, 5.0, 2.1)
**9'b**		1.99 m	41.5	2.21, td (13.6, 6.9)
**10'**	216.6	-	215.8	
**11'**	51.5	2.50 q(6.6)	50.3	2.56. q (6.7)
**11'CH₃**	8.8	0.68 d(6.6)	8.0	0.80, d (6.8)

Figure S1: MS/HRMS spectra (10, 20 and 40eV) for ascochlorin N-acetylglucosamine (**19**).

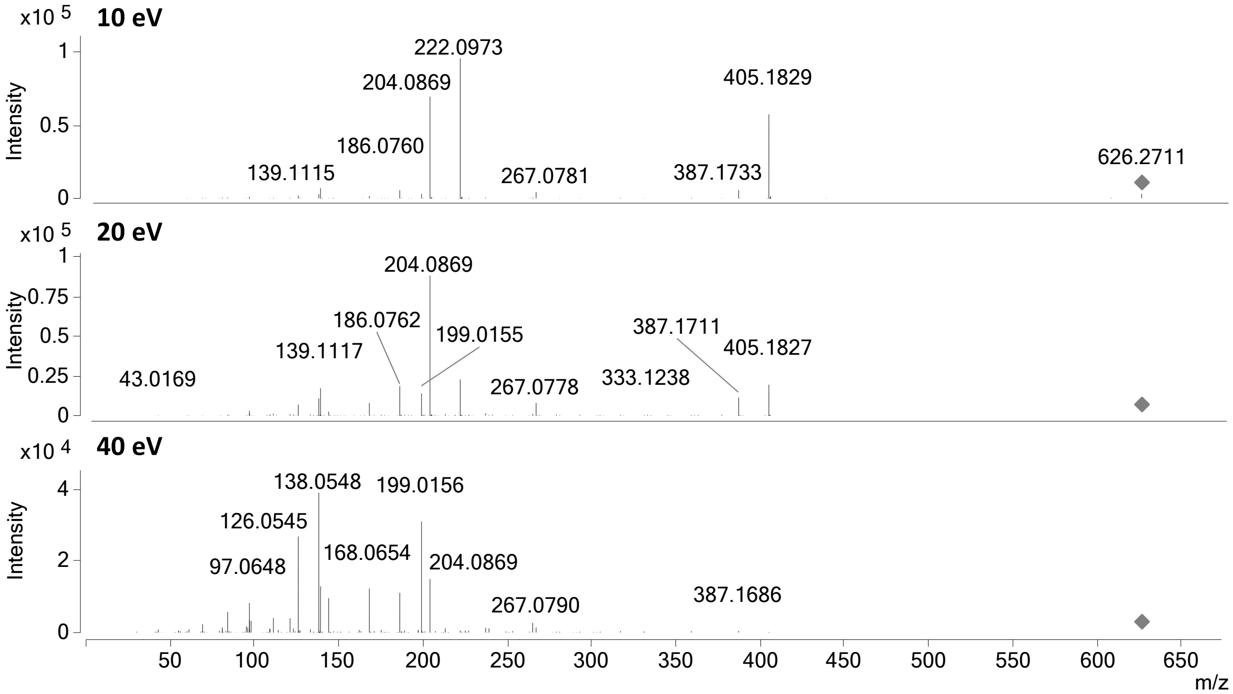

Figure S2: MS/HRMS spectra (10, 20 and 40eV) for 4'-ketoascochlorin (**20**).

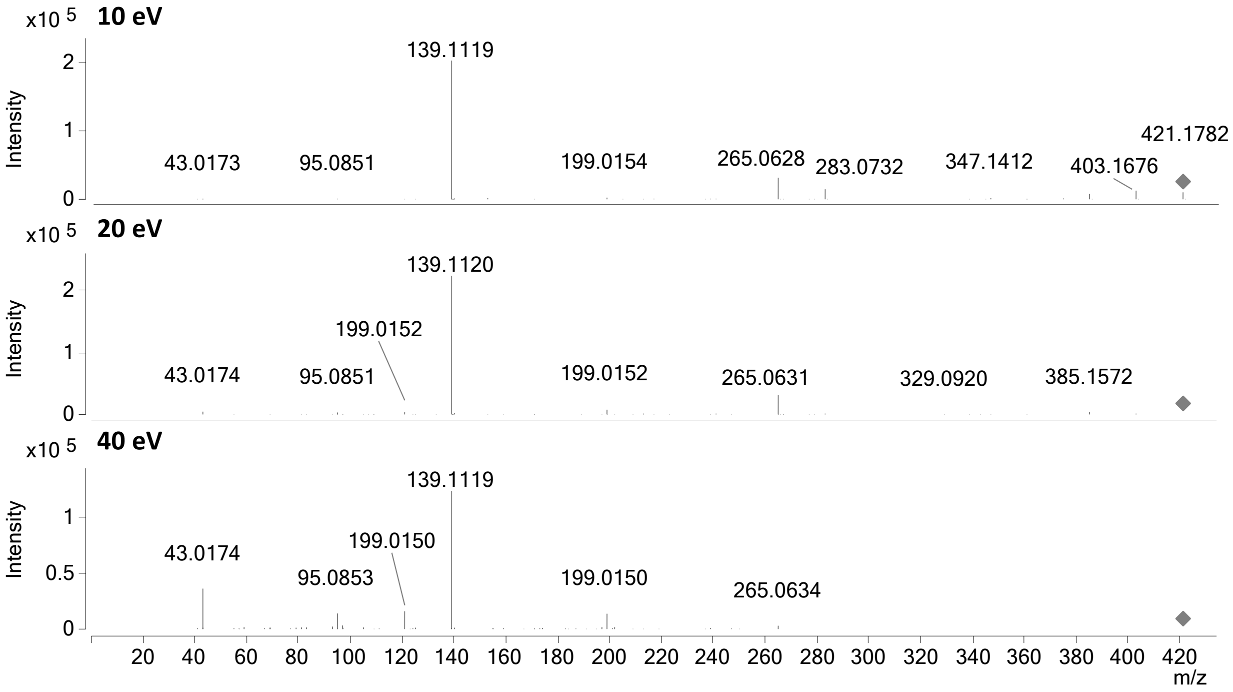

Figure S3: MS/HRMS spectra (10, 20 and 40eV) of $[M\text{-}CH_2O_2\text{+}H]^+$ for 4',5'- dihydro-4'-formylascochlorin (**21**). $[M\text{+}H]^+$ adduct was not observed.

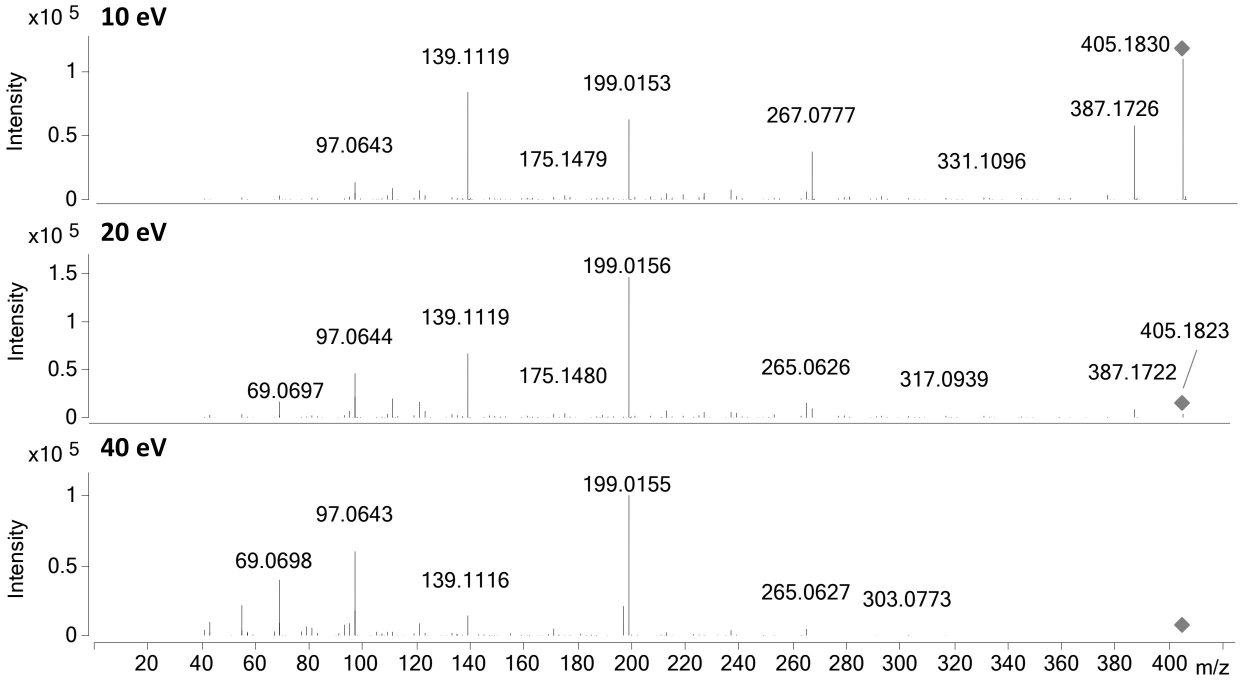

Figure S4: MS/HRMS spectra (10, 20 and 40eV) for fimetarin A (**22**).

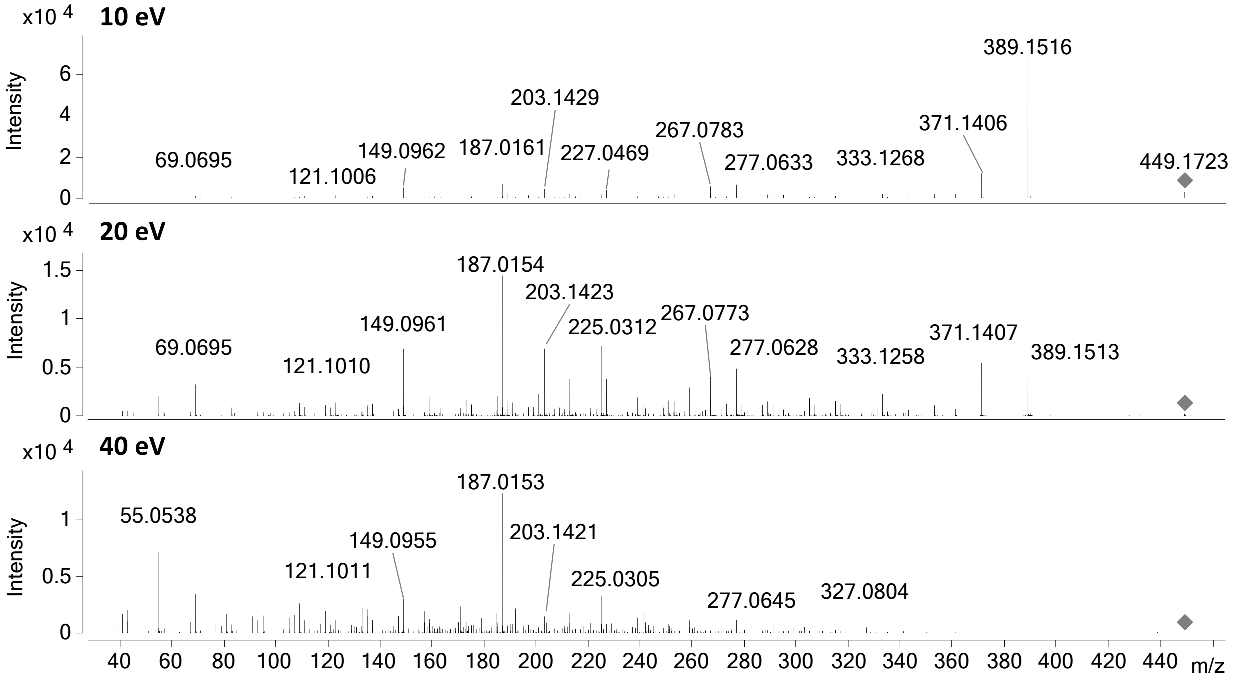

Figure S5: MS/HRMS spectra (10, 20 and 40eV) for fimetarin B (**23**).

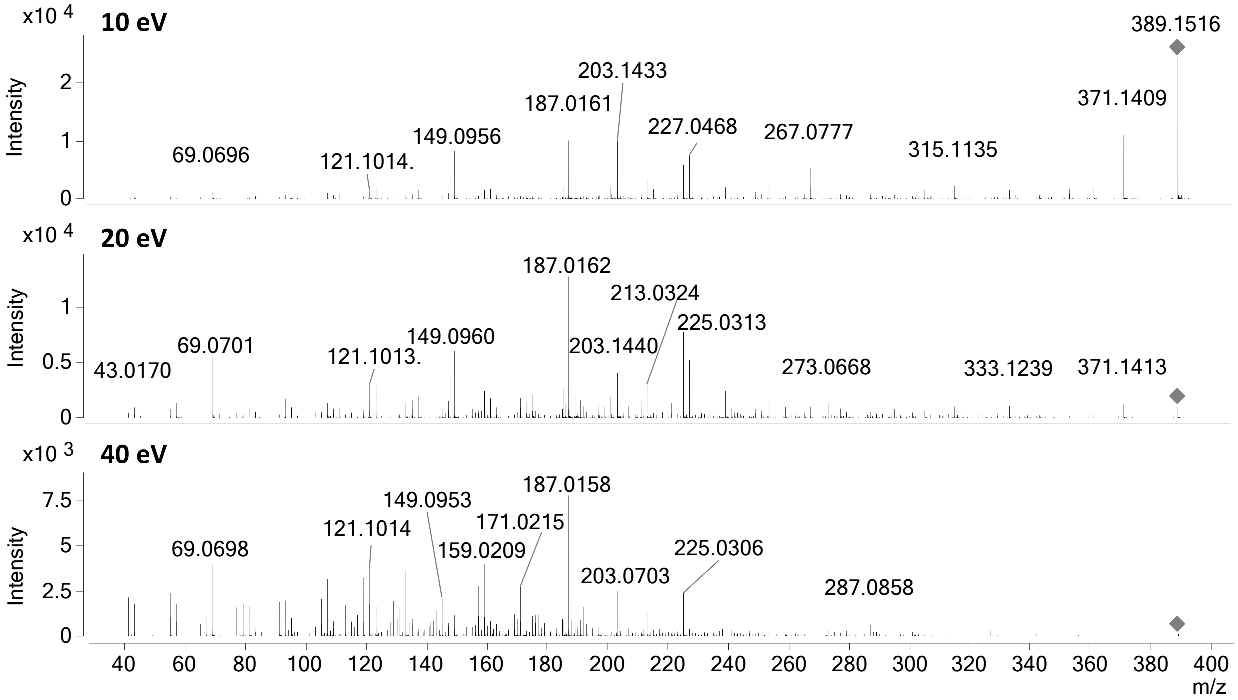

Figure S6: MS/HRMS spectra (10, 20 and 40eV) for fimetarin C (**24**).

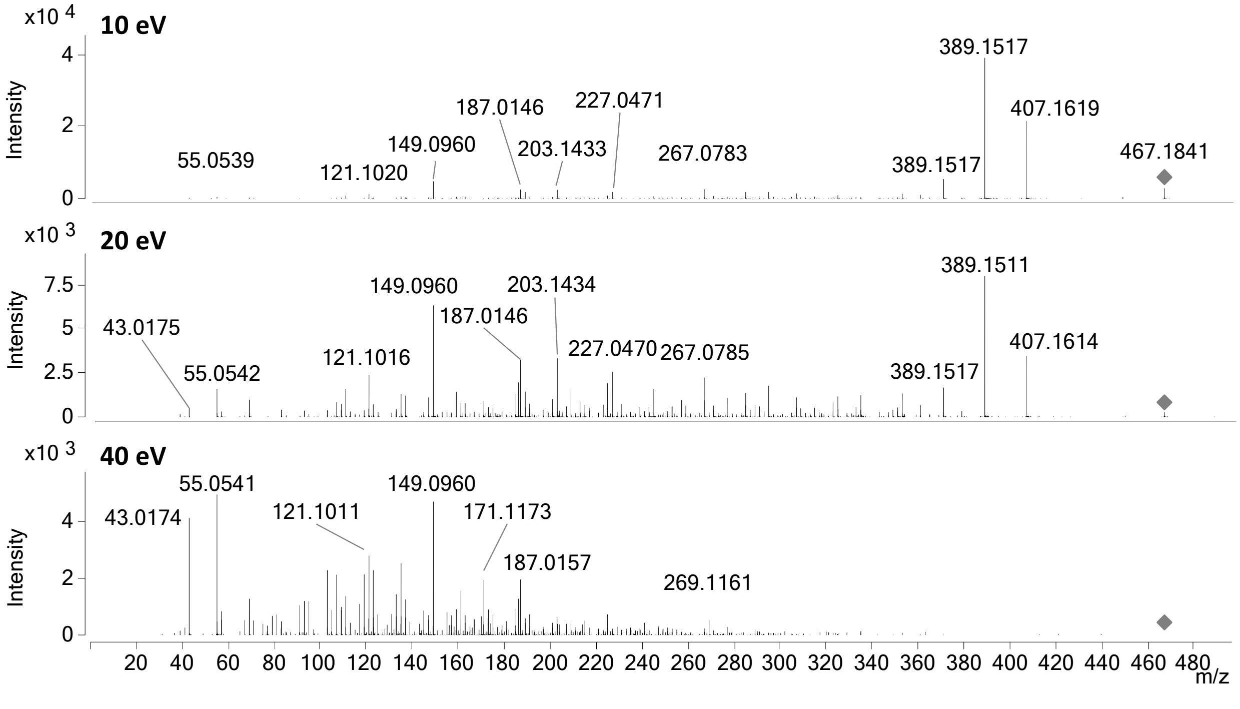

Figure S7: MS/HRMS spectra (10, 20 and 40eV) for fimetarin D (**25**).

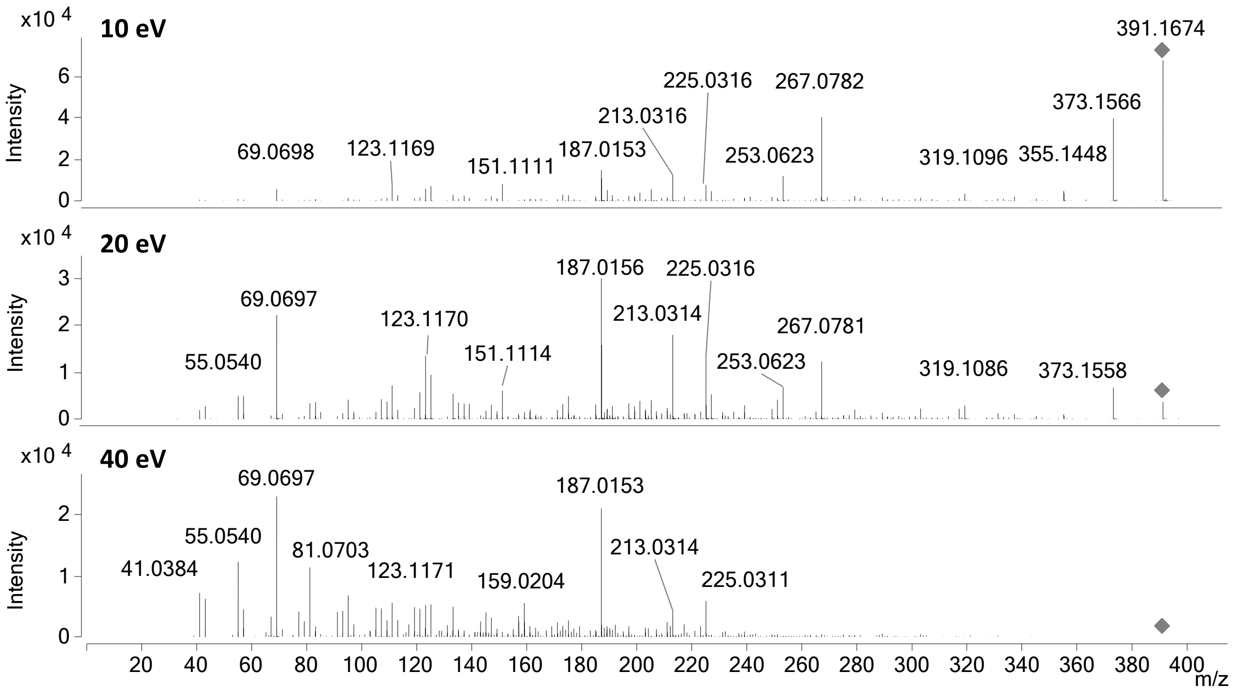

Figure S8. Proposed fragmentation patterns for fimetarins A-D (**22-25**) based on key fragments observed in 10 eV MS/HRMS spectra (Figures S4-S7). To the left, fragmentation patterns for fimetarins A-C (**22-24**), to the right – for fimetarin D (**25**). Fragments highlighted in green are observed in all four compounds, in blue – observed only in fimetarins A-C, in red – observed only in fimetarin D.

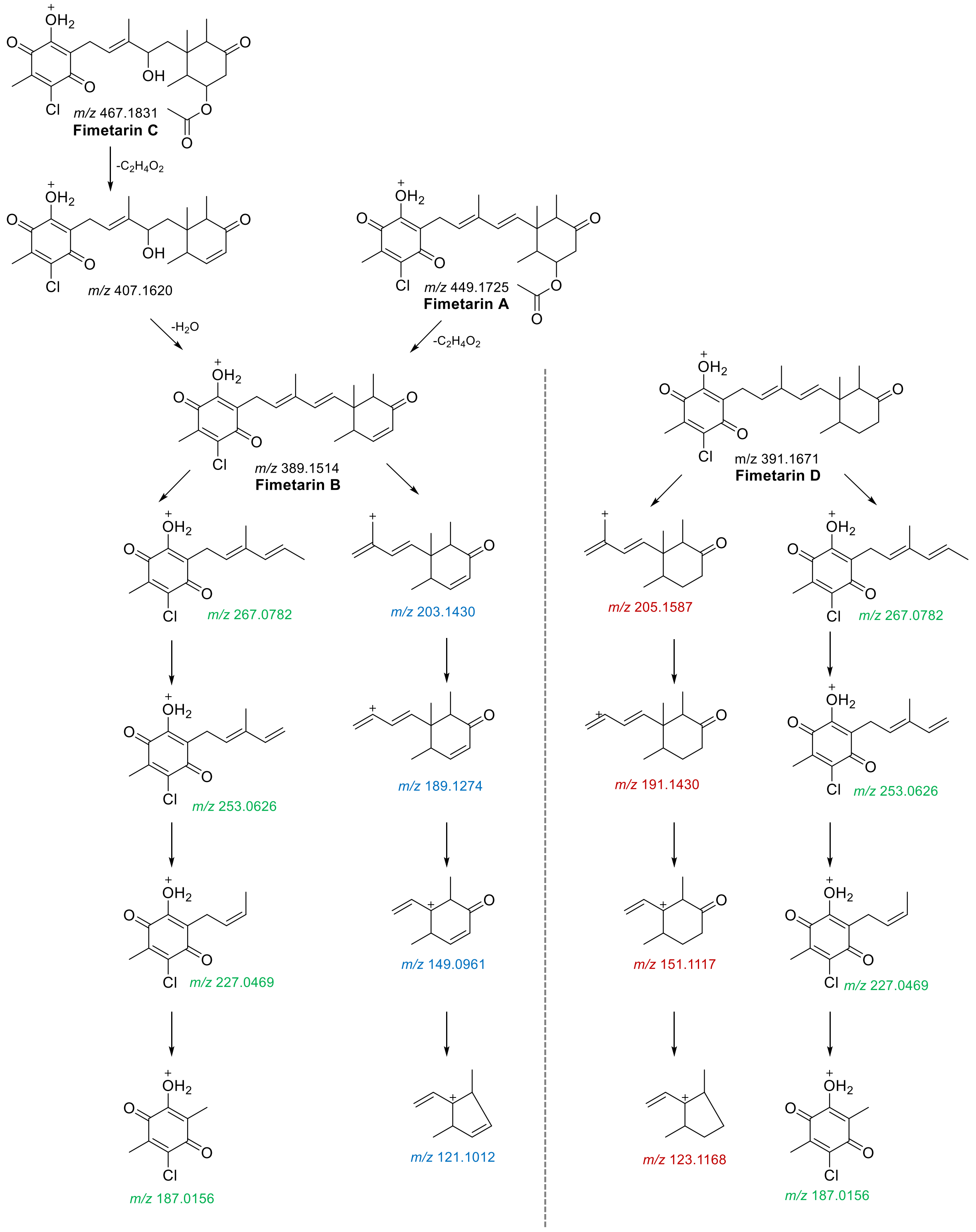

Figure S9. ^{1}H and HSQC spectra for ilicicolin D (**1**) in $CDCl_3$.

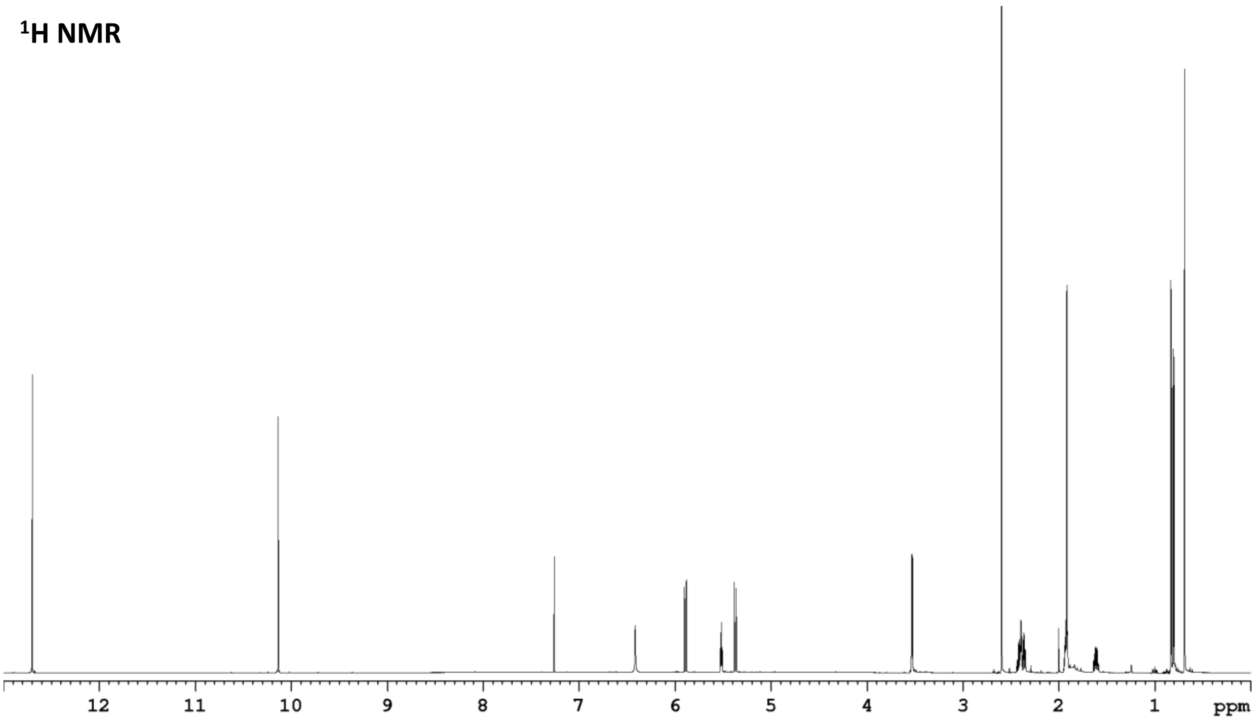

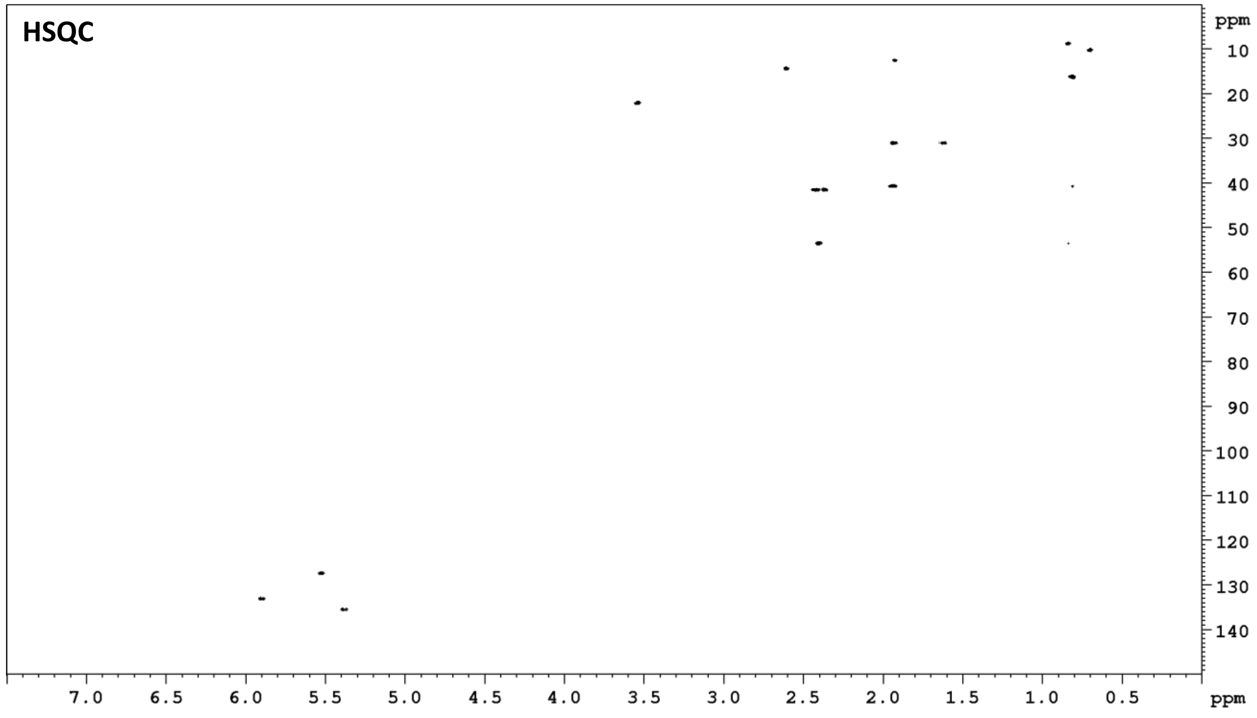

Figure S10. ^1H and HSQC spectra for ilicicolin F (**3**) in $CDCl_3$.

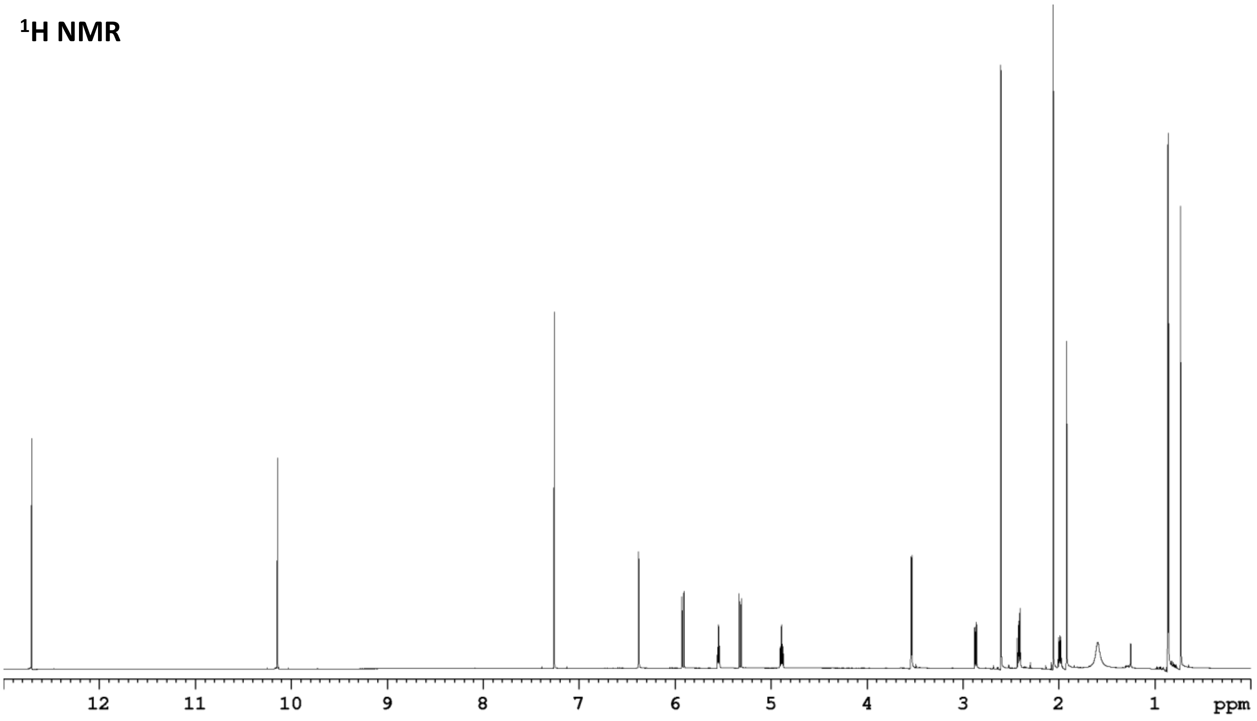

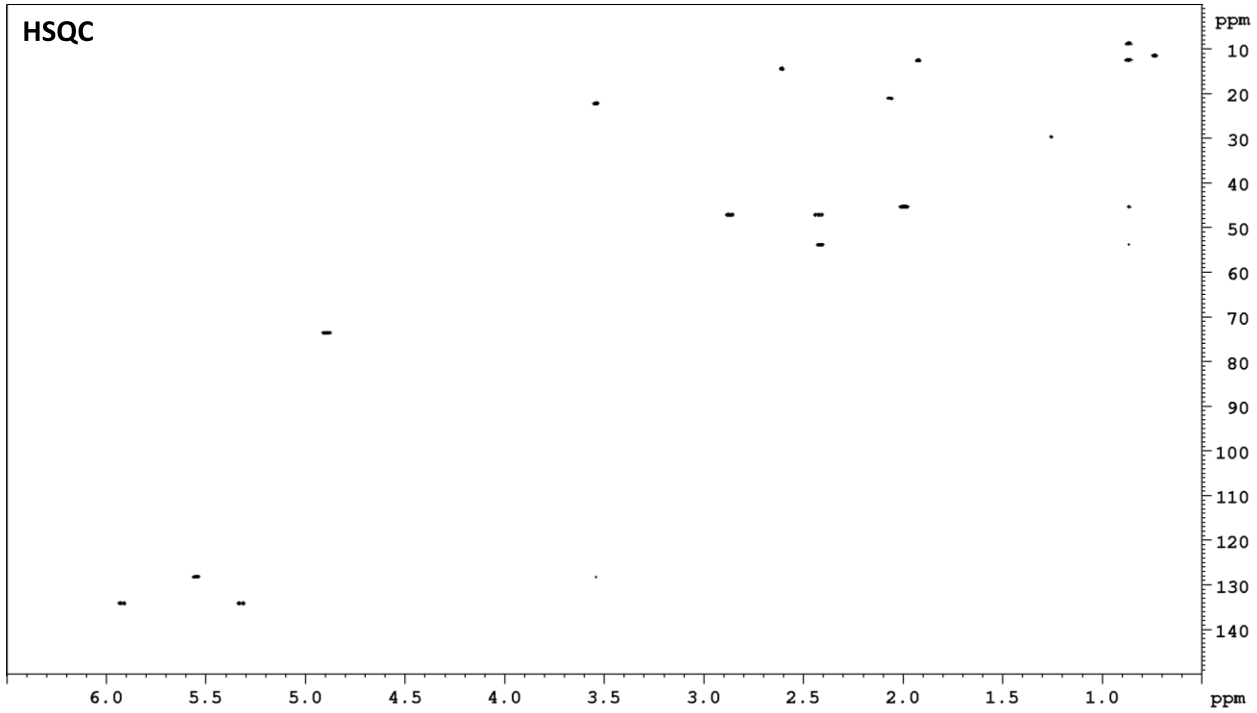

Figure S11. ^1H and HSQC spectra for ilicicolin C (**5**) in $CDCl_3$.

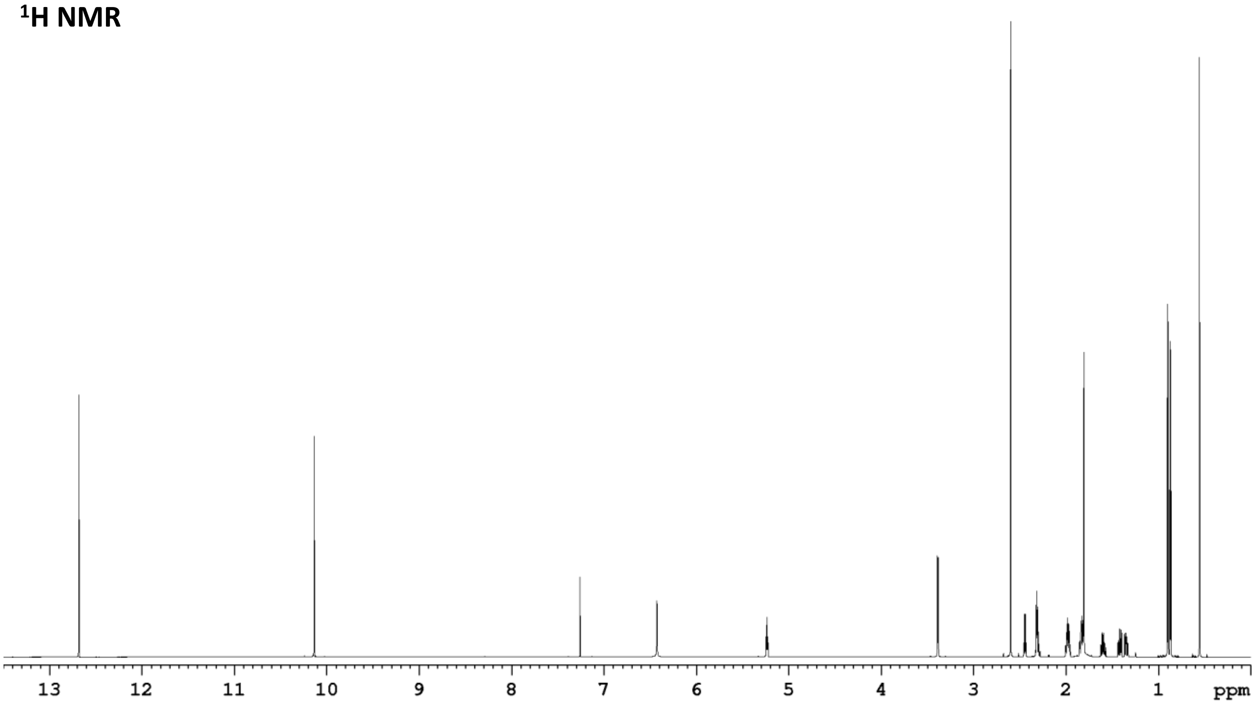

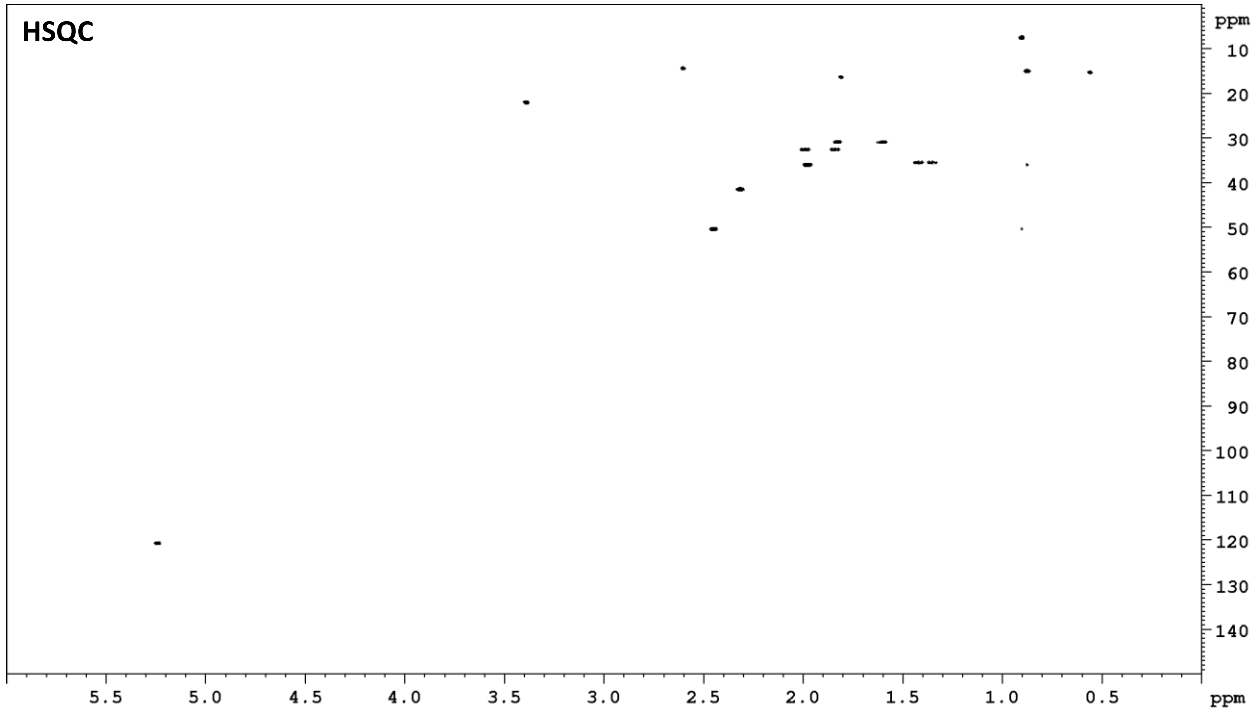

Figure S12. 1D and 2D NMR spectra for 4’5’-dihydro-4’-hydroxyascochlorin (**6**) in CD_3OD.

^{1}H NMR

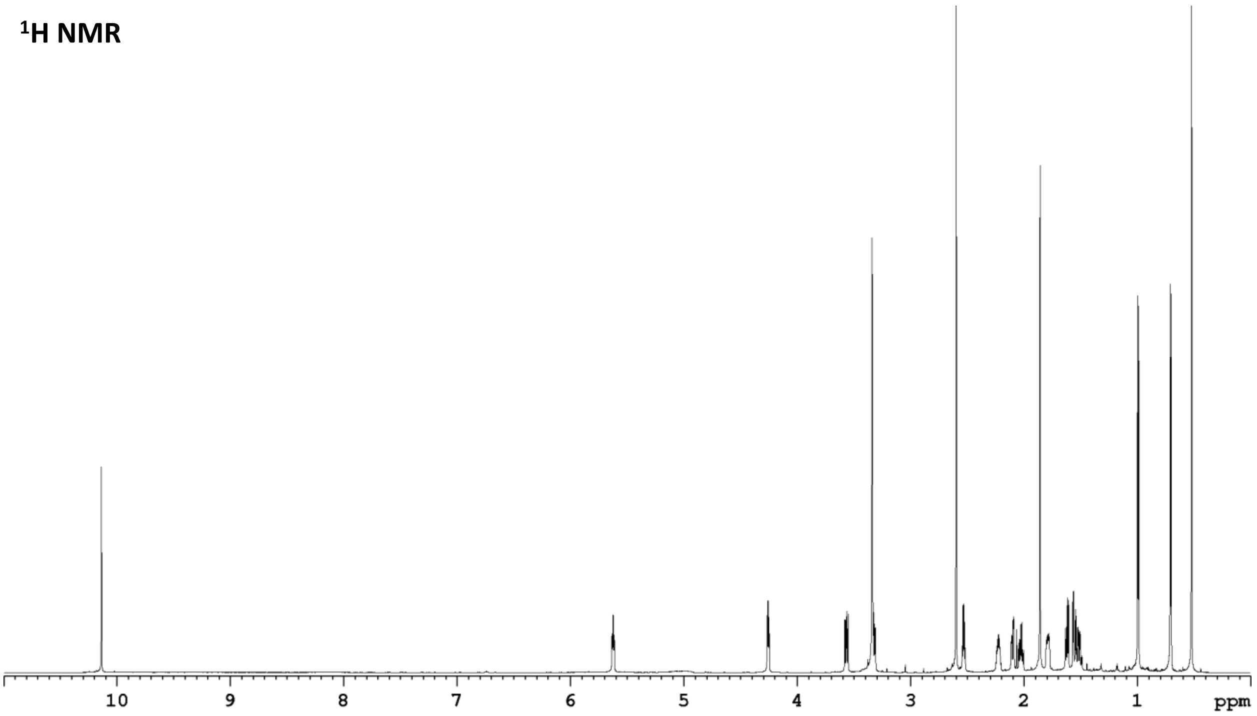

^{13}C NMR

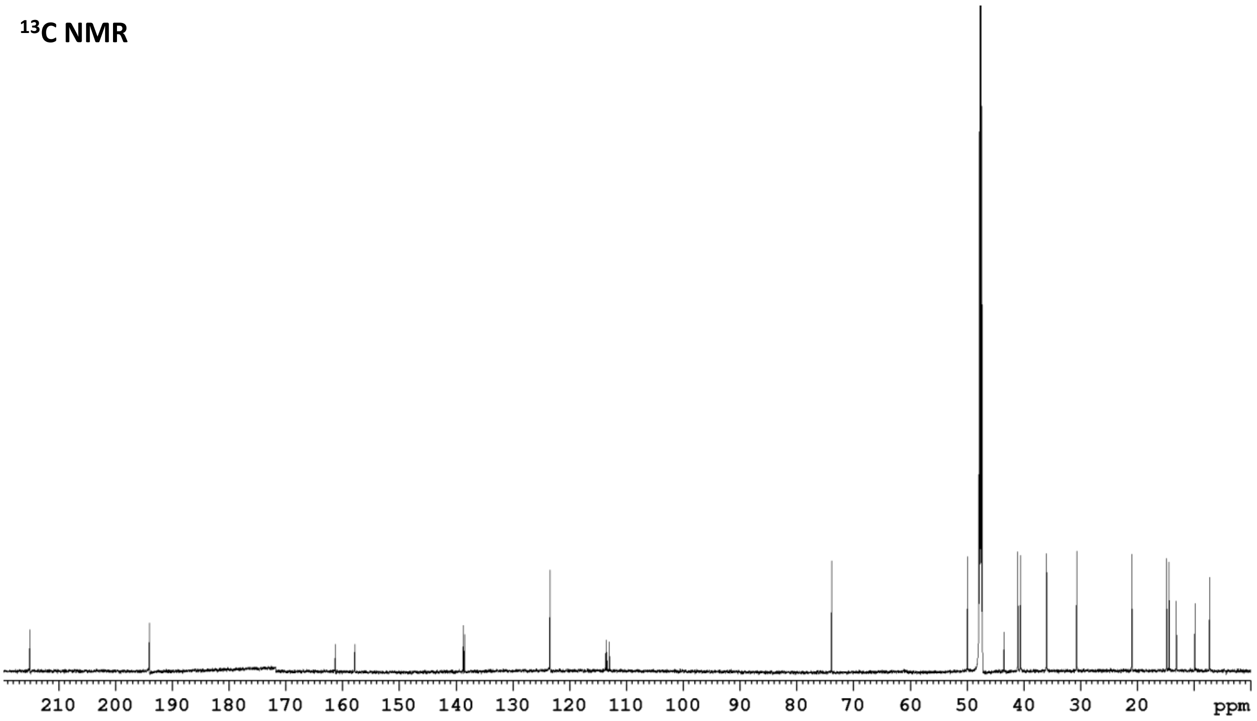

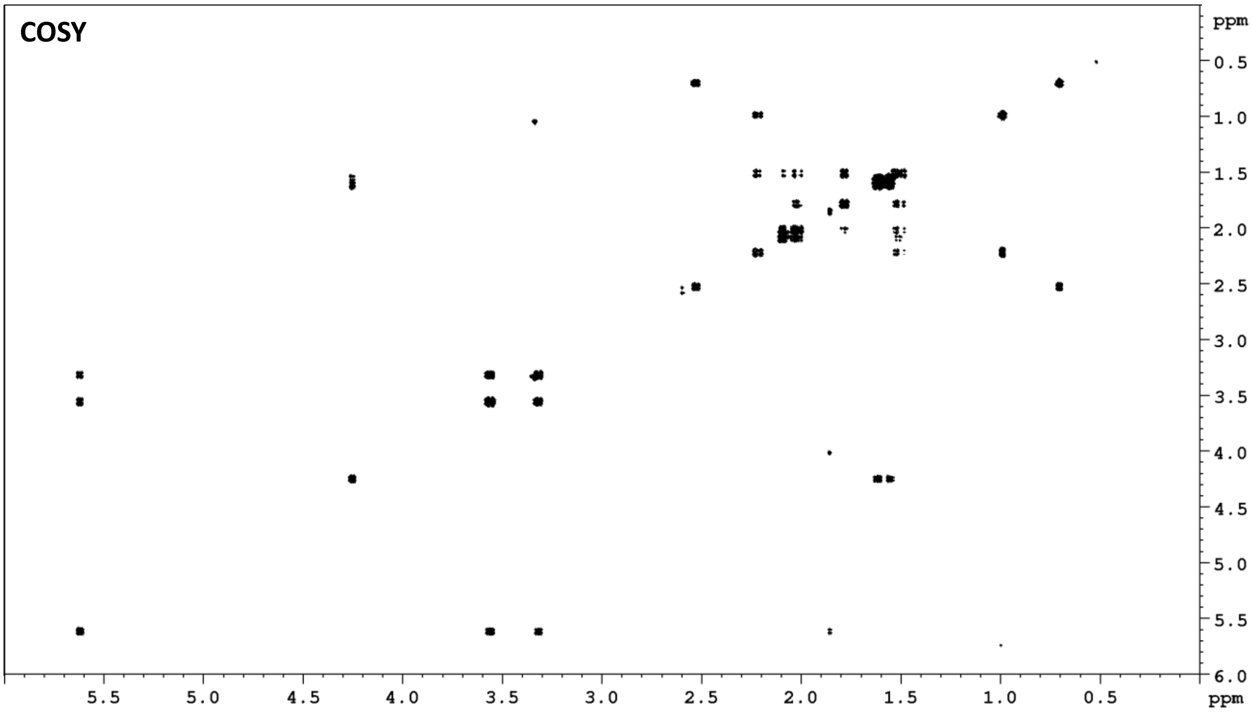
COSY
ppm
0.5
1.0
1.5
2.0
2.5
3.0
3.5
4.0
4.5
5.0
5.5
6.0
5.5
5.0
4.5
4.0
3.5
3.0
2.5
2.0
1.5
1.0
0.5
ppm

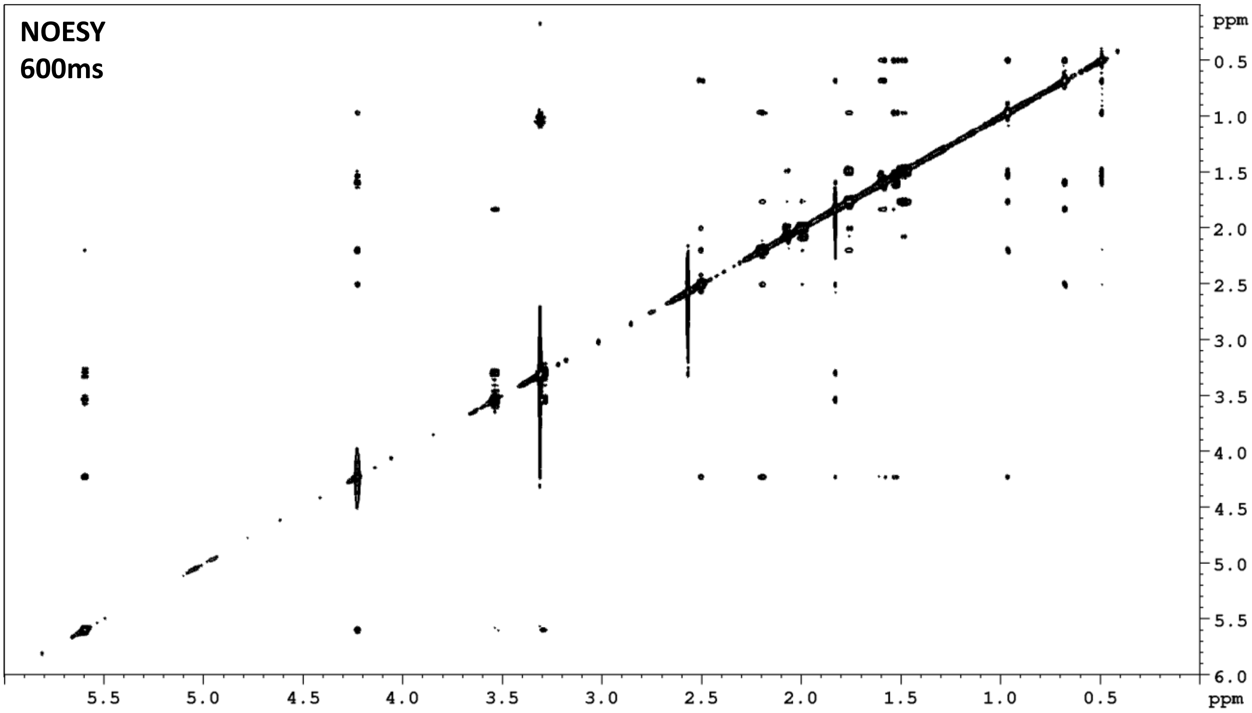
NOESY
600ms
ppm
0.5
1.0
1.5
2.0
2.5
3.0
3.5
4.0
4.5
5.0
5.5
6.0
5.5
5.0
4.5
4.0
3.5
3.0
2.5
2.0
1.5
1.0
0.5
ppm

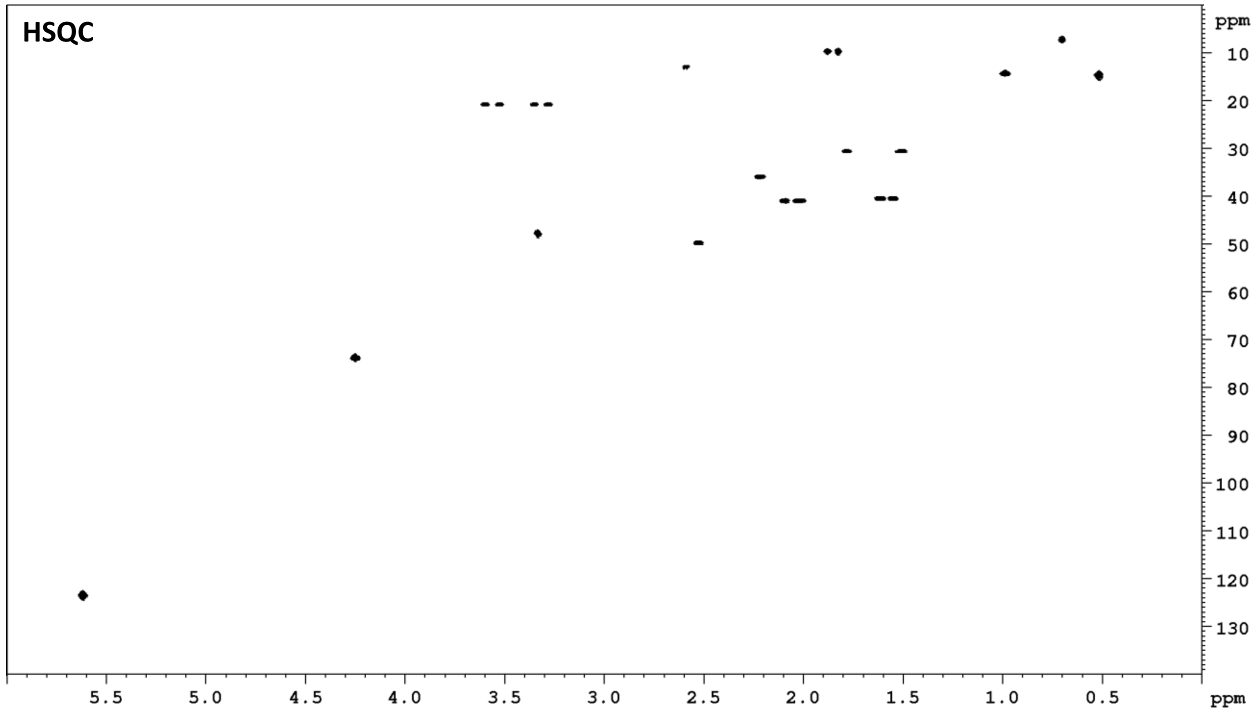
HSQC
ppm
10
20
30
40
50
60
70
80
90
100
110
120
130
5.5
5.0
4.5
4.0
3.5
3.0
2.5
2.0
1.5
1.0
0.5
ppm

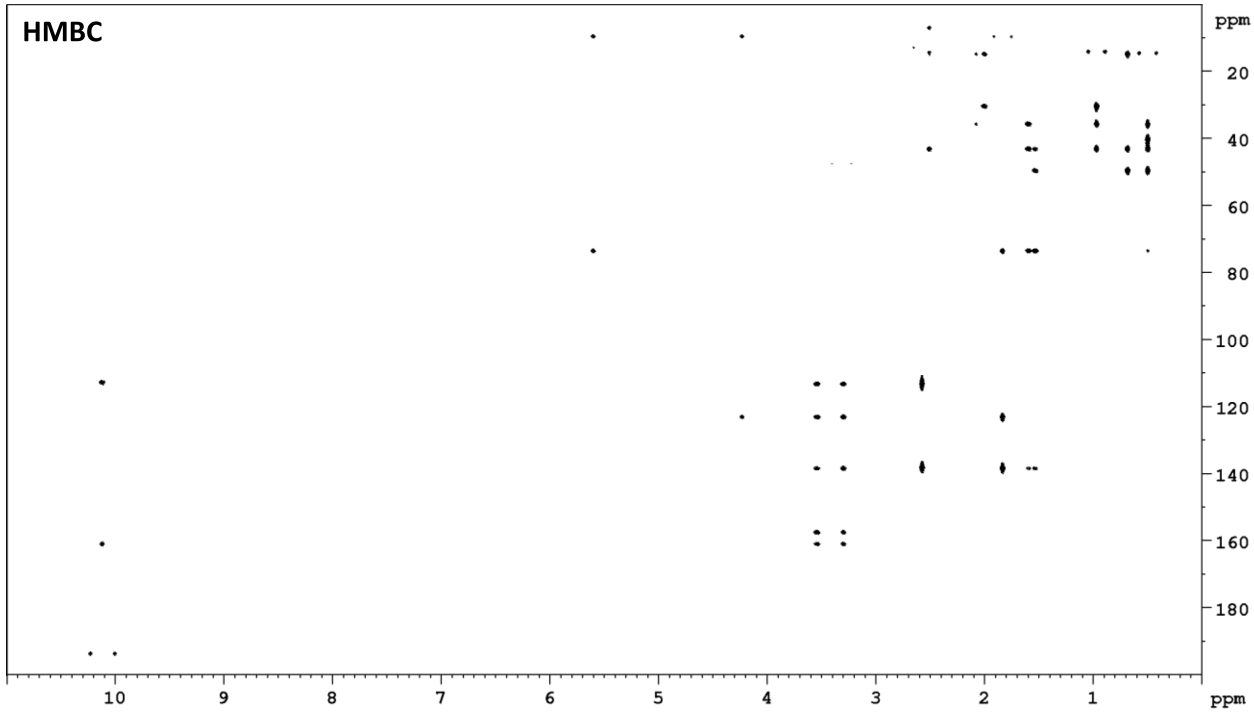
HMBC
ppm
20
40
60
80
100
120
140
160
180
10
9
8
7
6
5
4
3
2
1
ppm

Figure S13. 1D and 2D NMR spectra for 4'5'-dihydro-4'-hydroxyascochlorin (**6**) in $CDCl_3$.

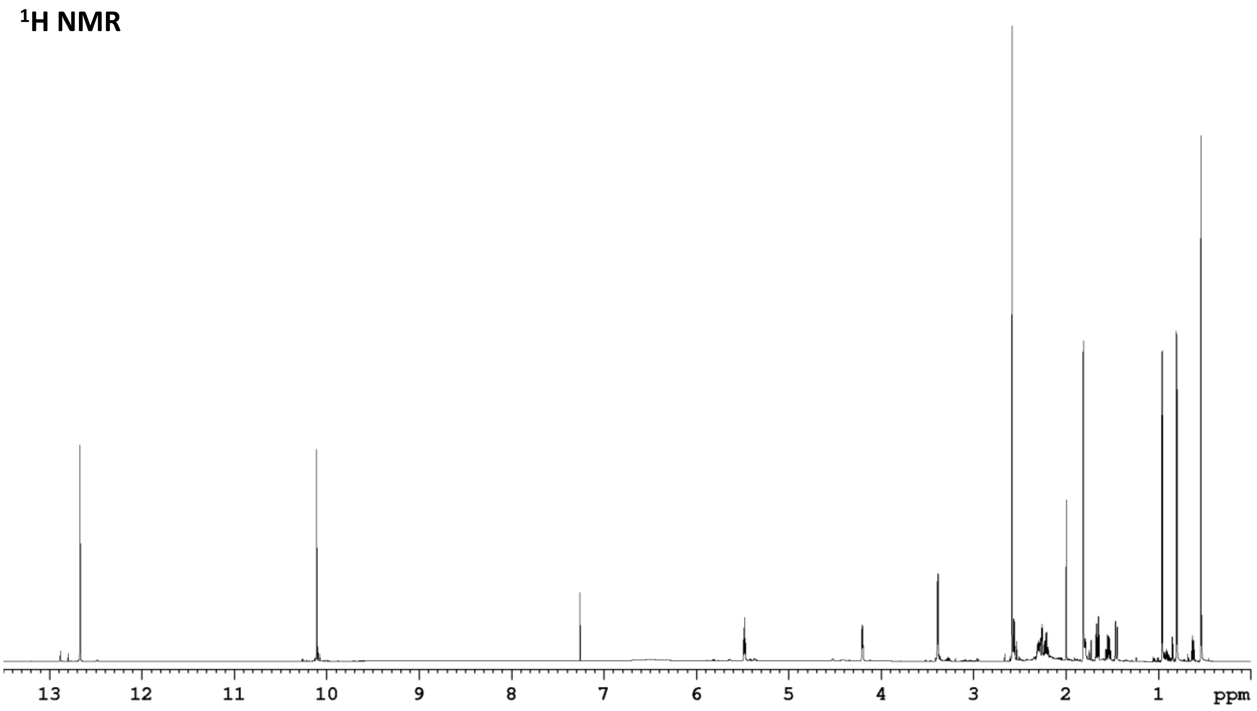

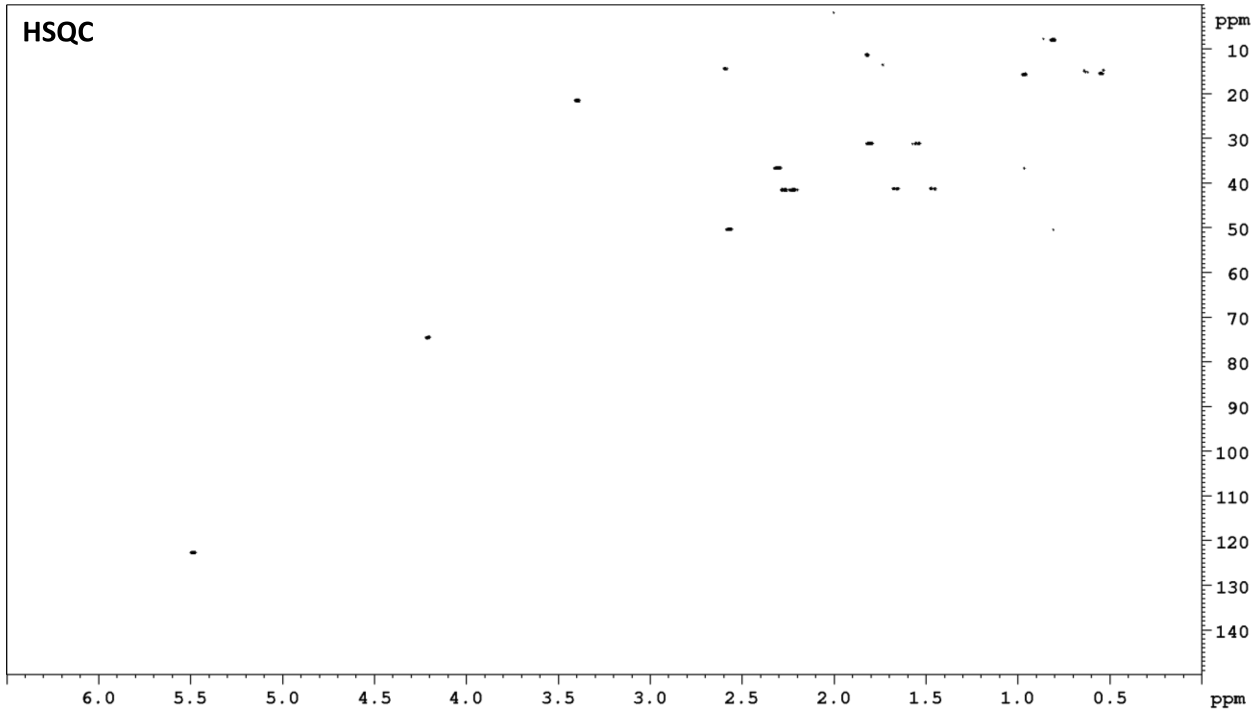

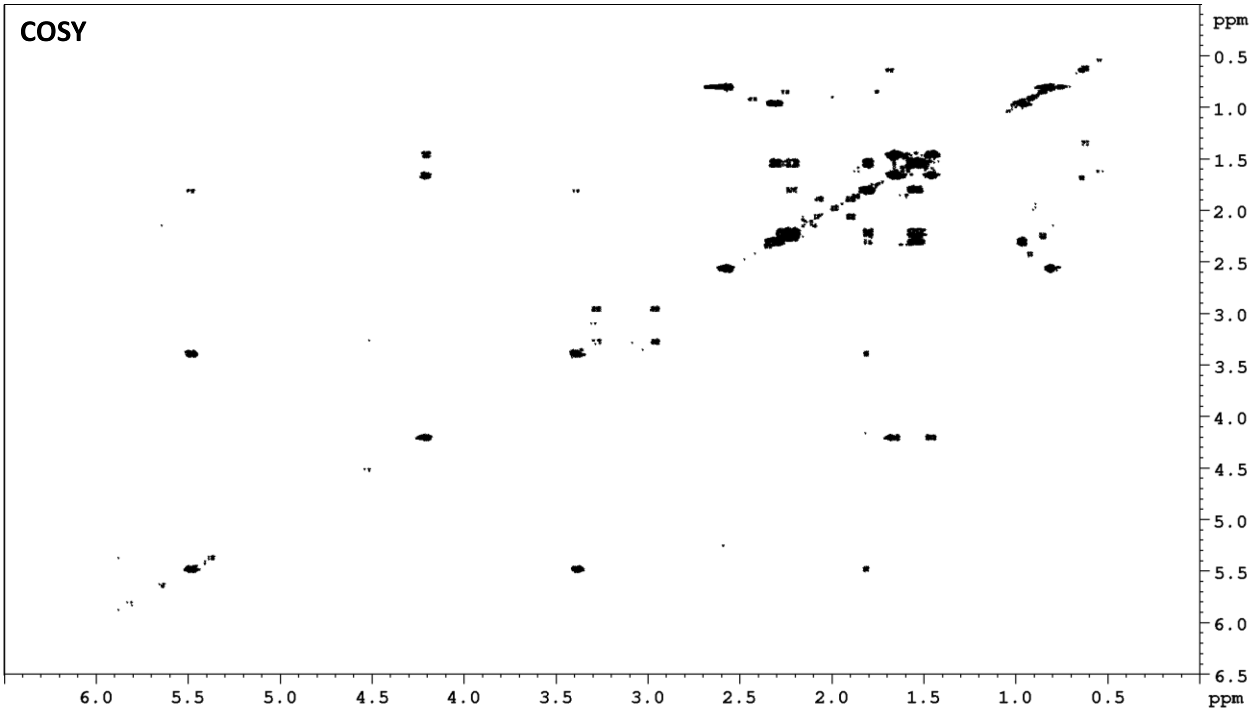
COSY
ppm
0.5
1.0
1.5
2.0
2.5
3.0
3.5
4.0
4.5
5.0
5.5
6.0
6.5
6.0
5.5
5.0
4.5
4.0
3.5
3.0
2.5
2.0
1.5
1.0
0.5
ppm

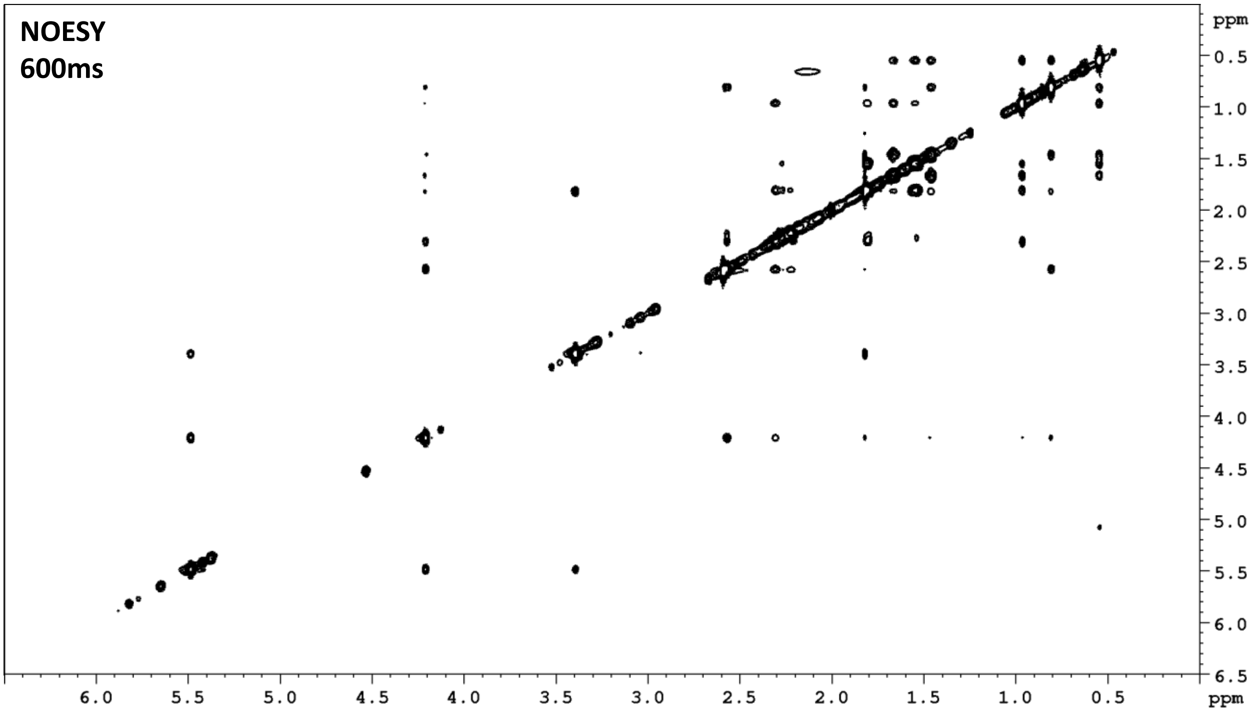
NOESY
600ms
ppm
0.5
1.0
1.5
2.0
2.5
3.0
3.5
4.0
4.5
5.0
5.5
6.0
6.5
6.0
5.5
5.0
4.5
4.0
3.5
3.0
2.5
2.0
1.5
1.0
0.5
ppm

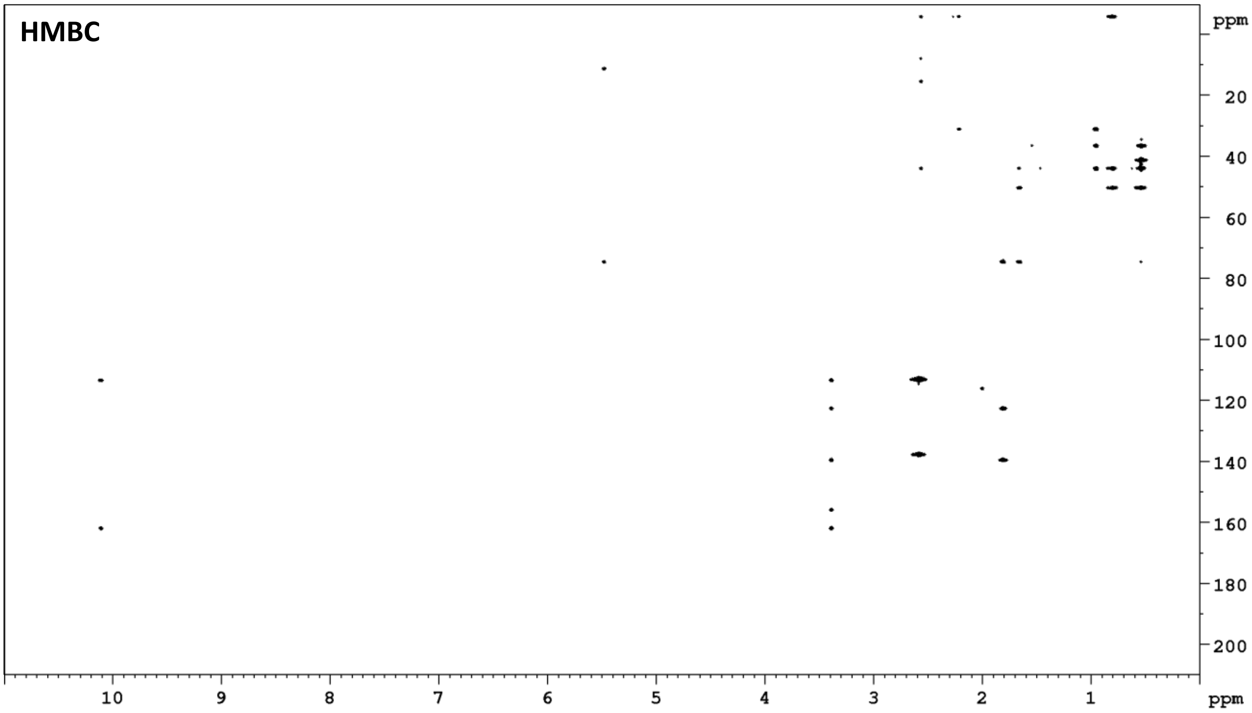

*The ketone group at C-10’ is observed at the wrong ^{13}C chemical shift (δ_C -5.8ppm) due to ^{13}C SW set to 220ppm.

Figure S14. ^{1}H and HSQC spectra for LL-Z1272ε (**8**) in $CDCl_3$.

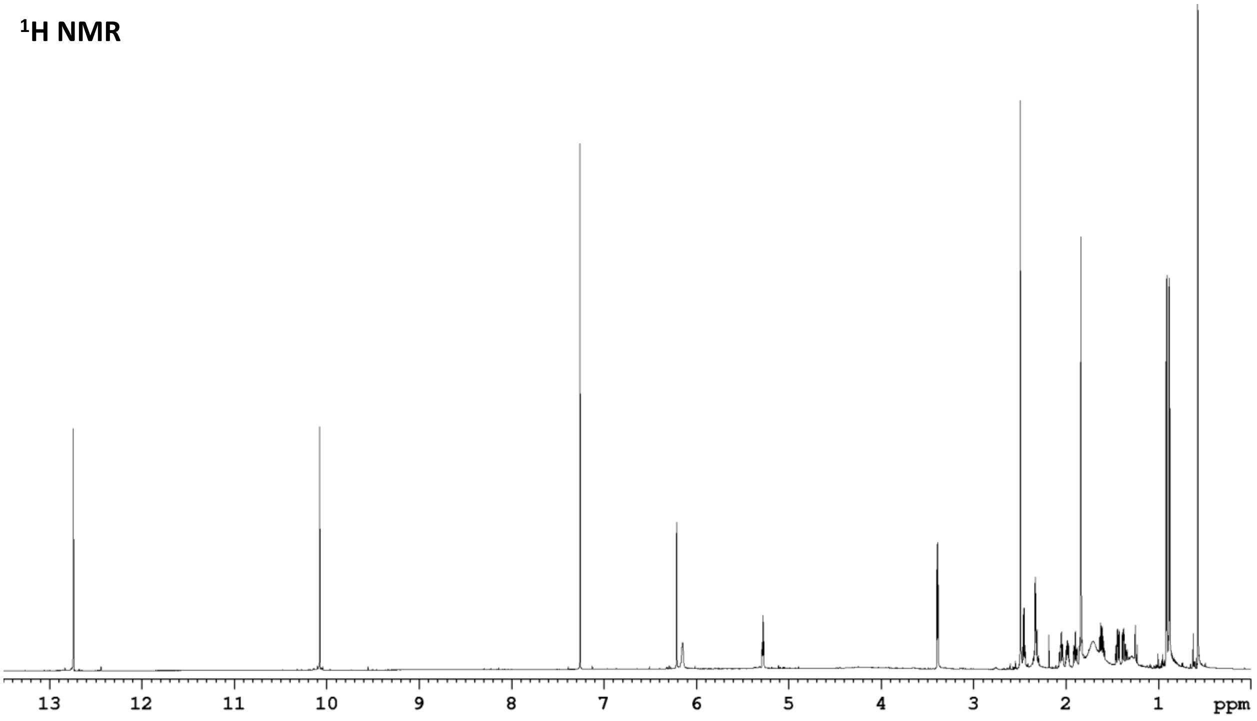

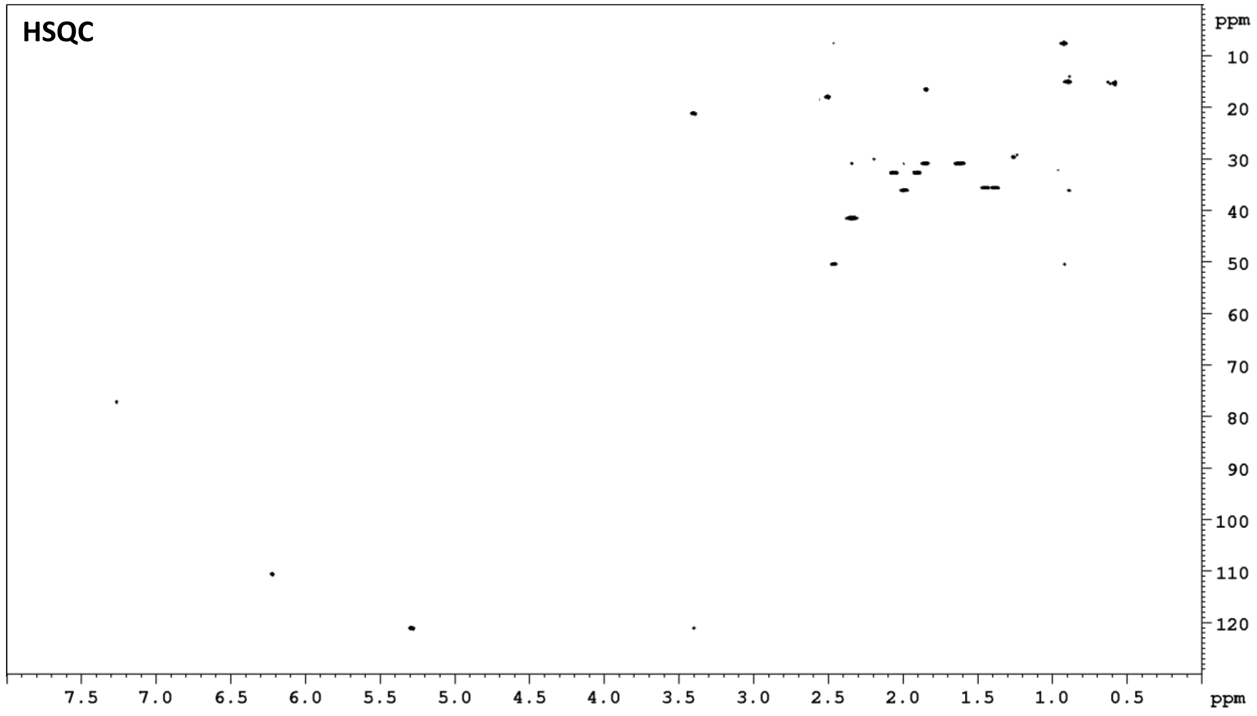

Figure S15. ^{1}H and HSQC spectra for ilicicolin E (**9**) in $CDCl_3$.

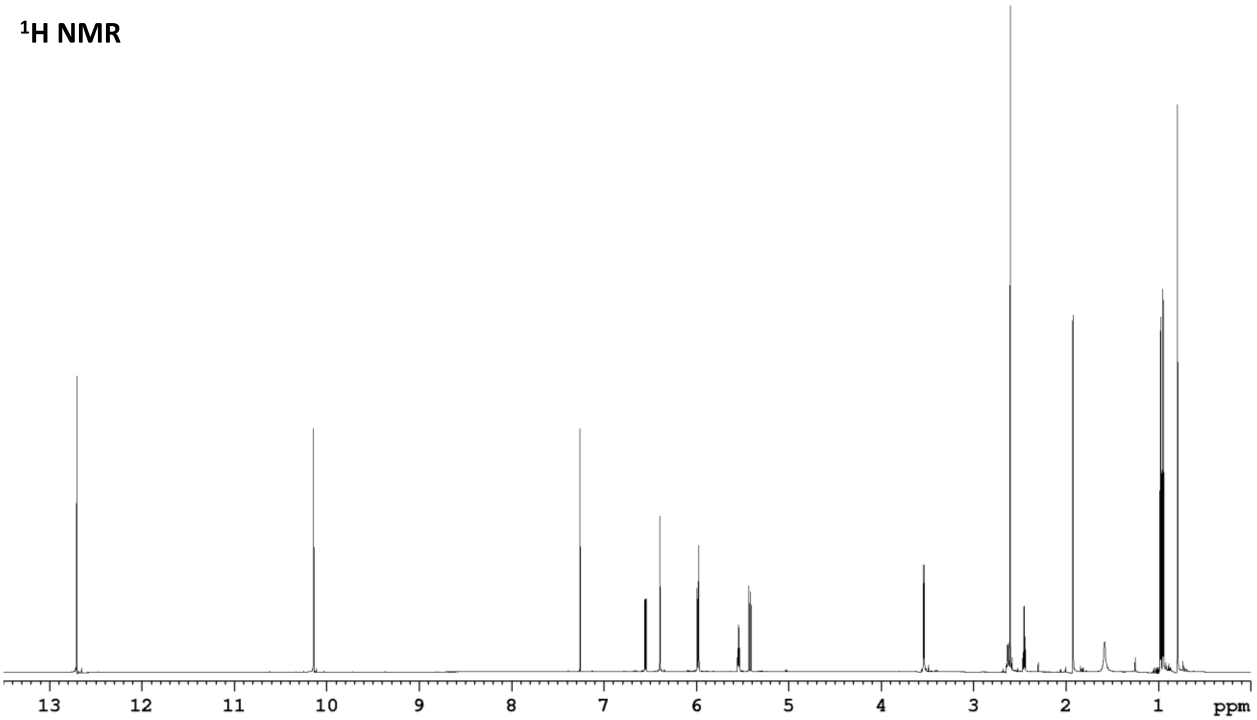

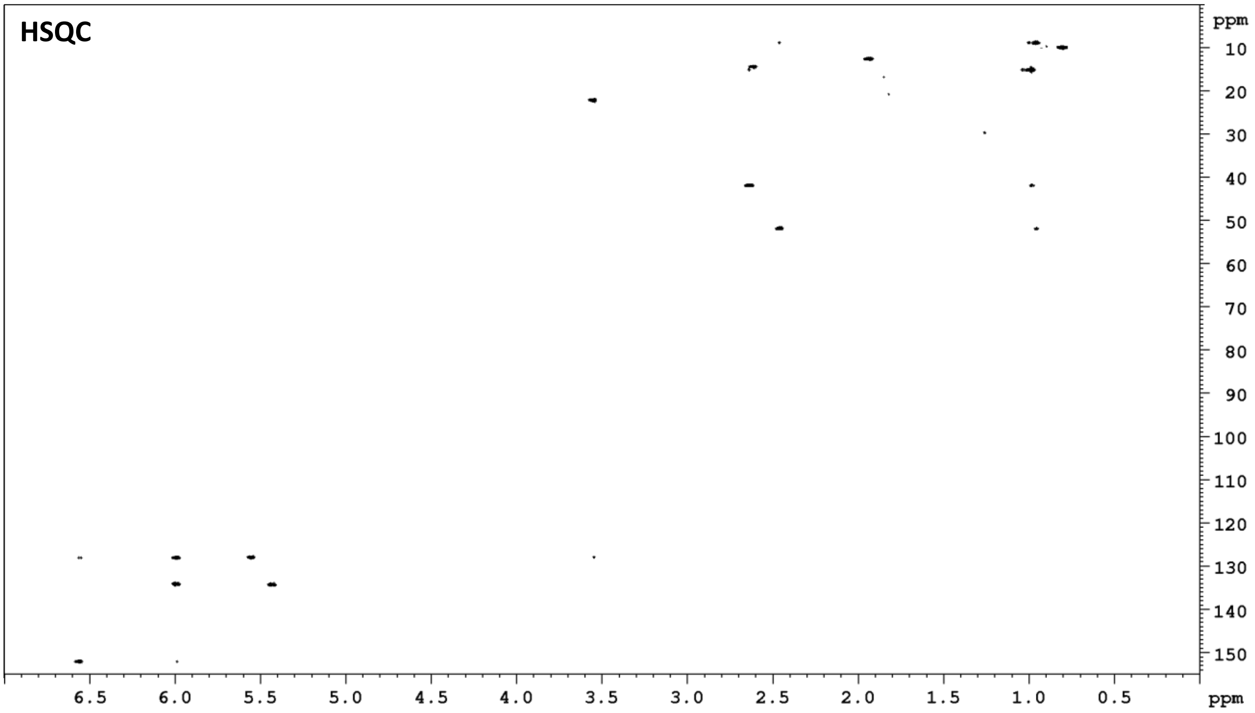

Figure S16. ^{1}H and HSQC spectra for ascofuranol (**10**) in $CDCl_3$.

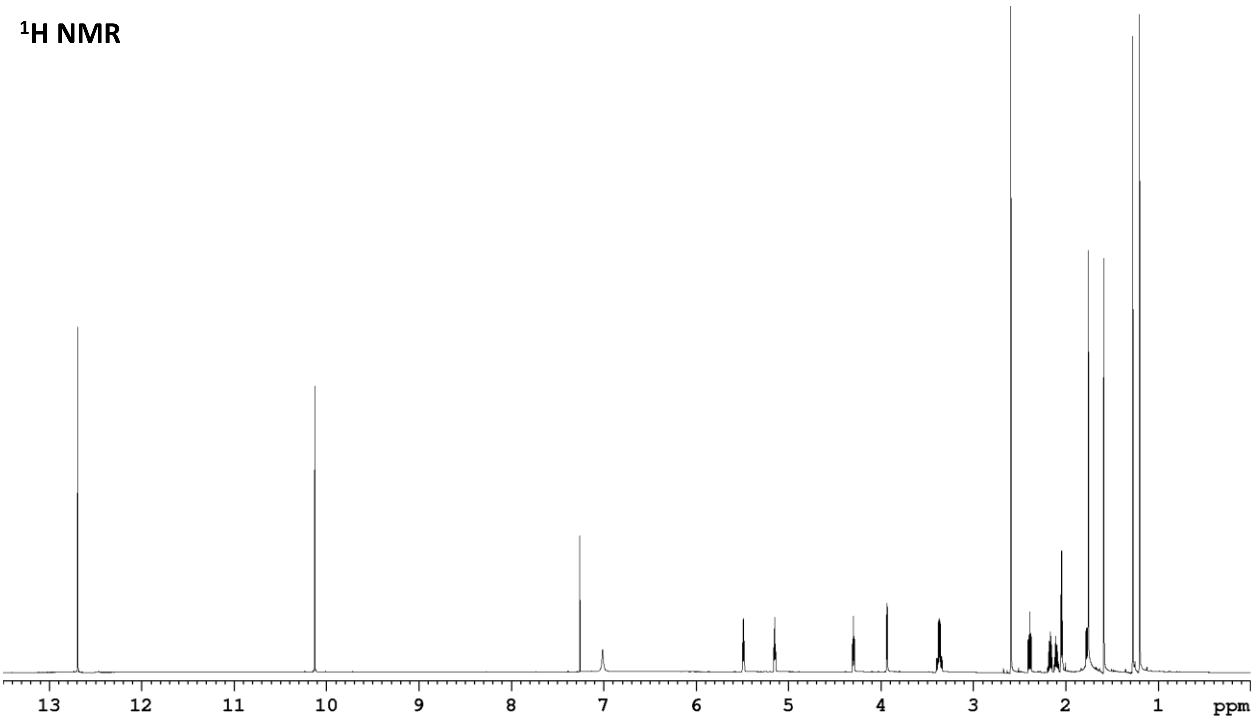

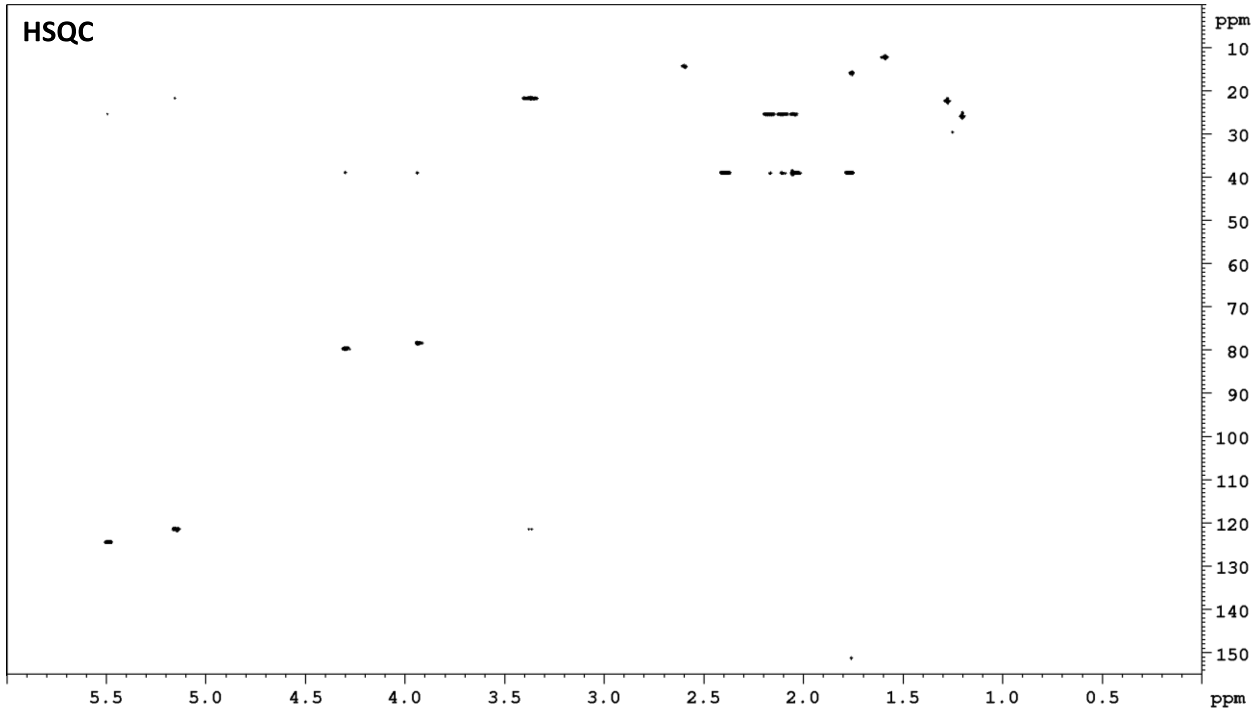

Figure S17. ^{1}H and HSQC spectra for ascofuranone (**11**) in $CDCl_3$.

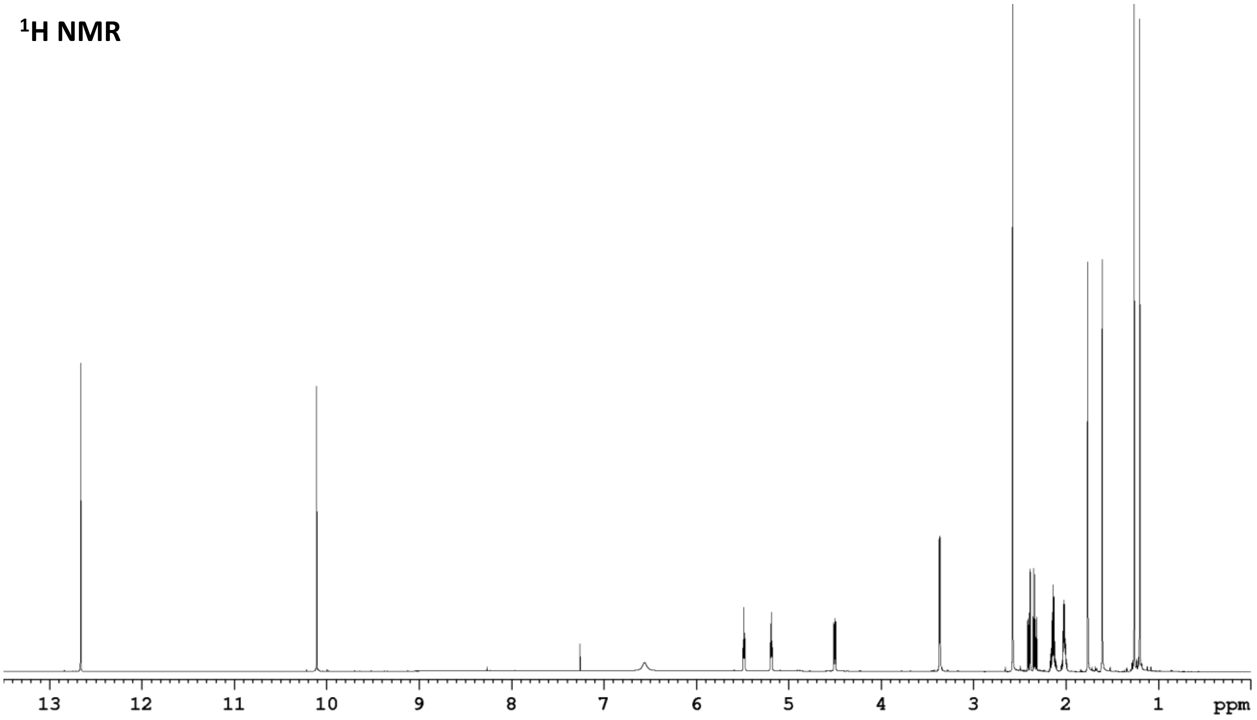

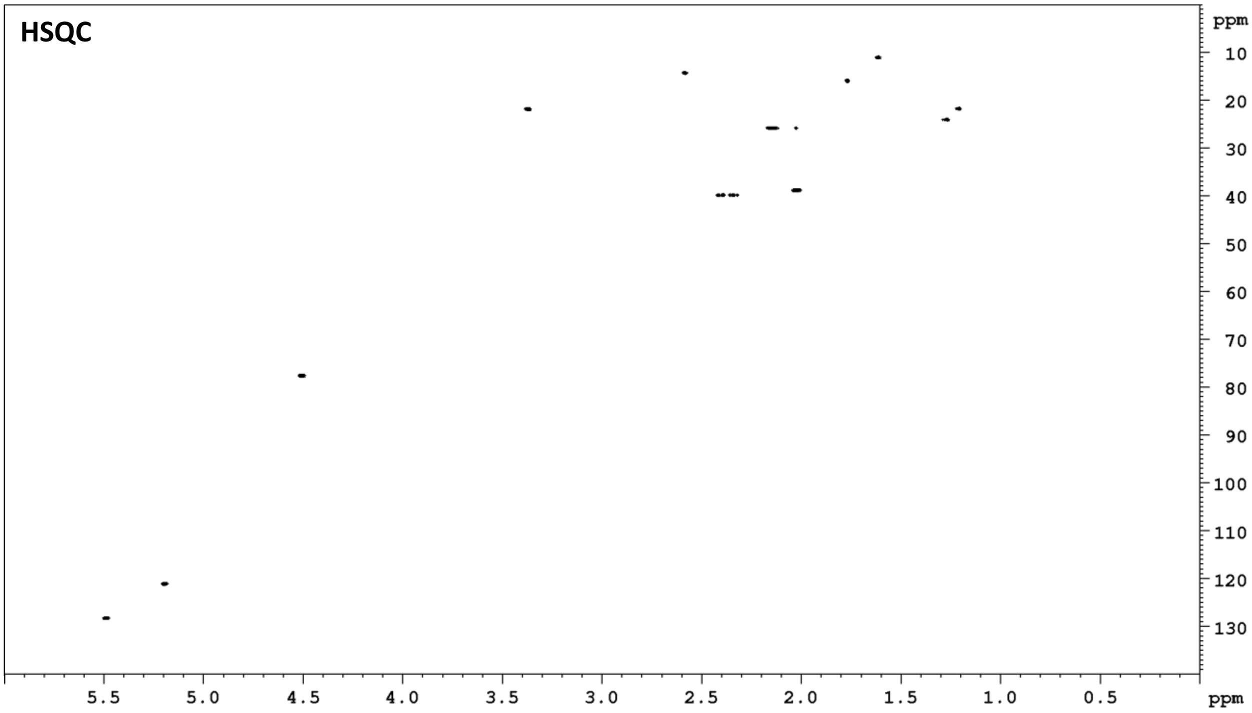

Figure S18. 1D and 2D NMR spectra for ascochlorin N-acetylglucosamine (**19**) in CD_3OD.

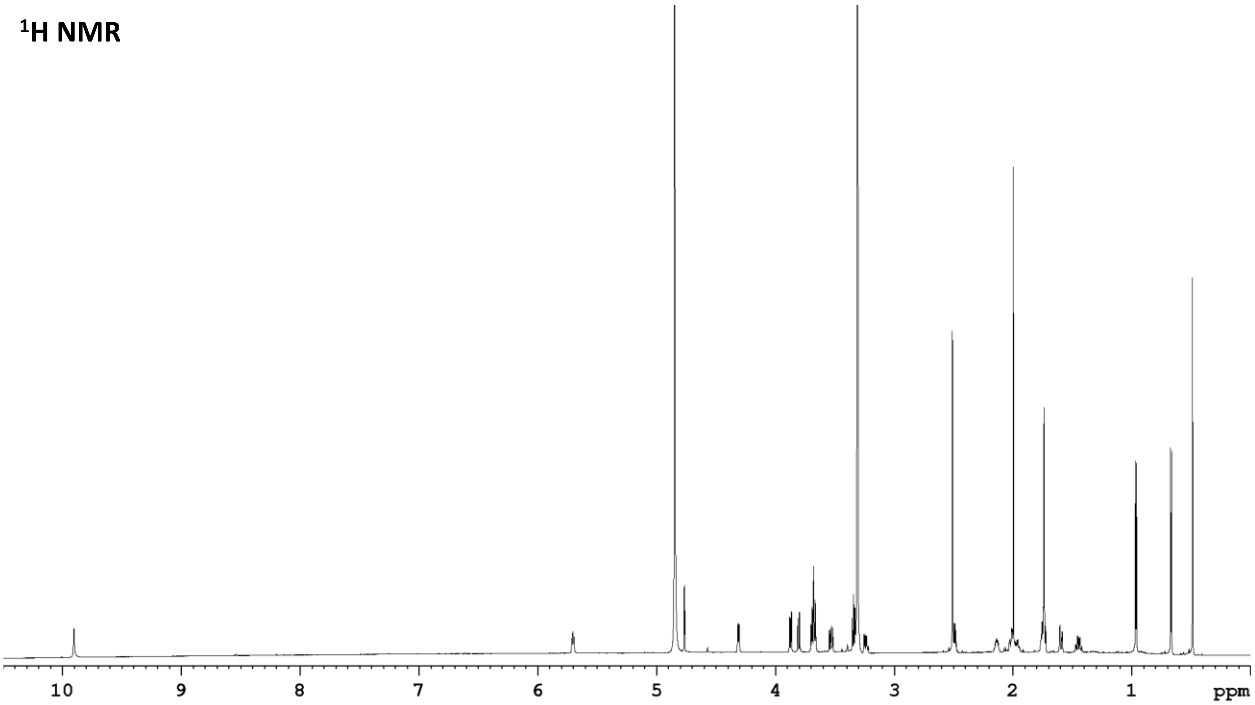

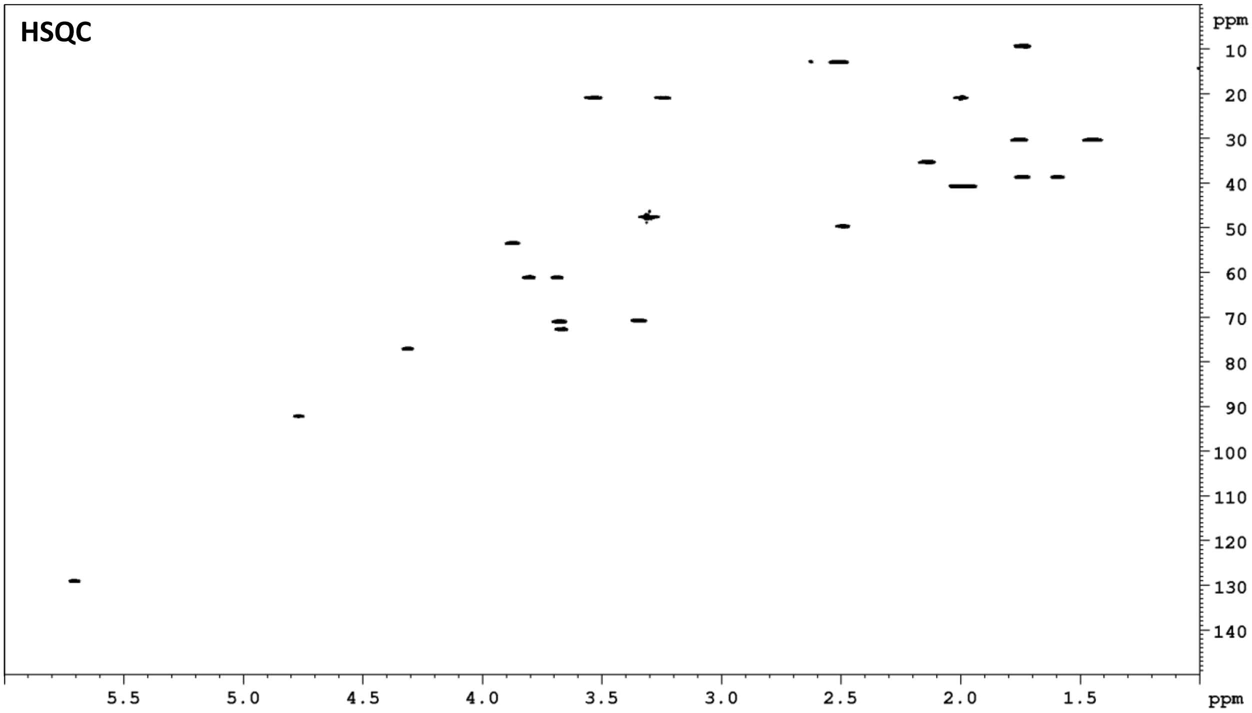

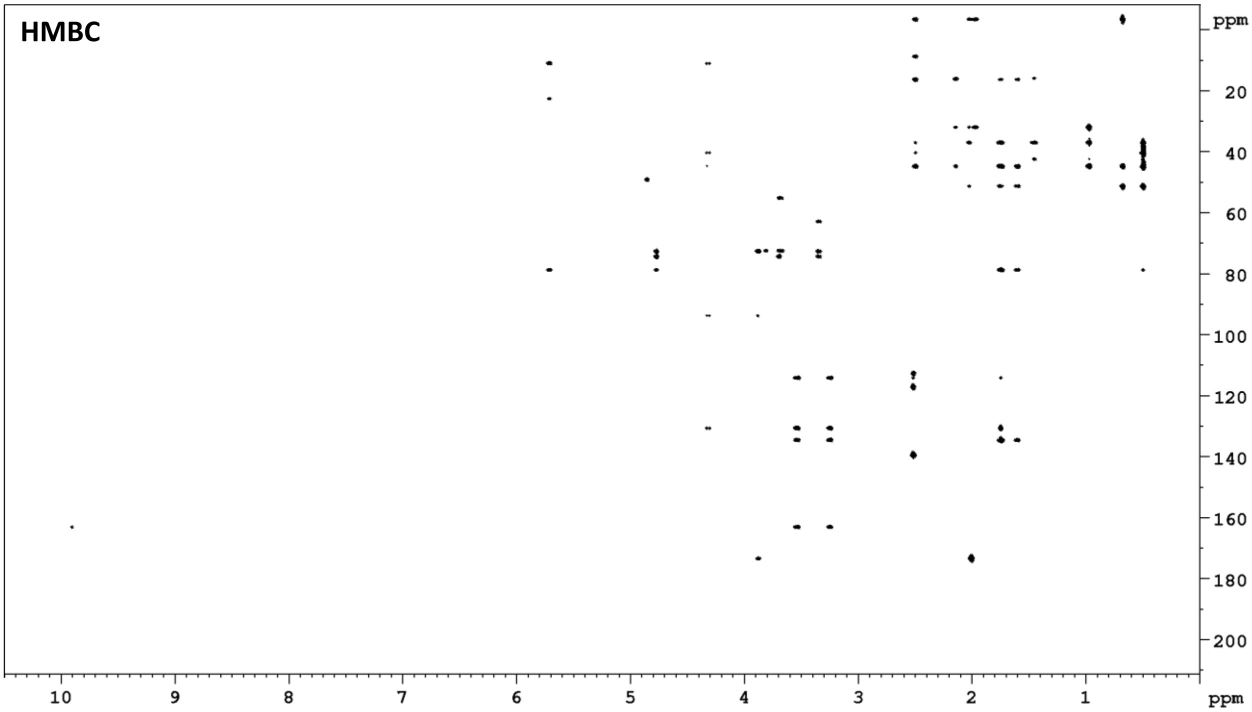

*The ketone group at C-10′ is observed at the wrong ^{13}C chemical shift (δ_C -3.3ppm) due to ^{13}C SW set to 220ppm.

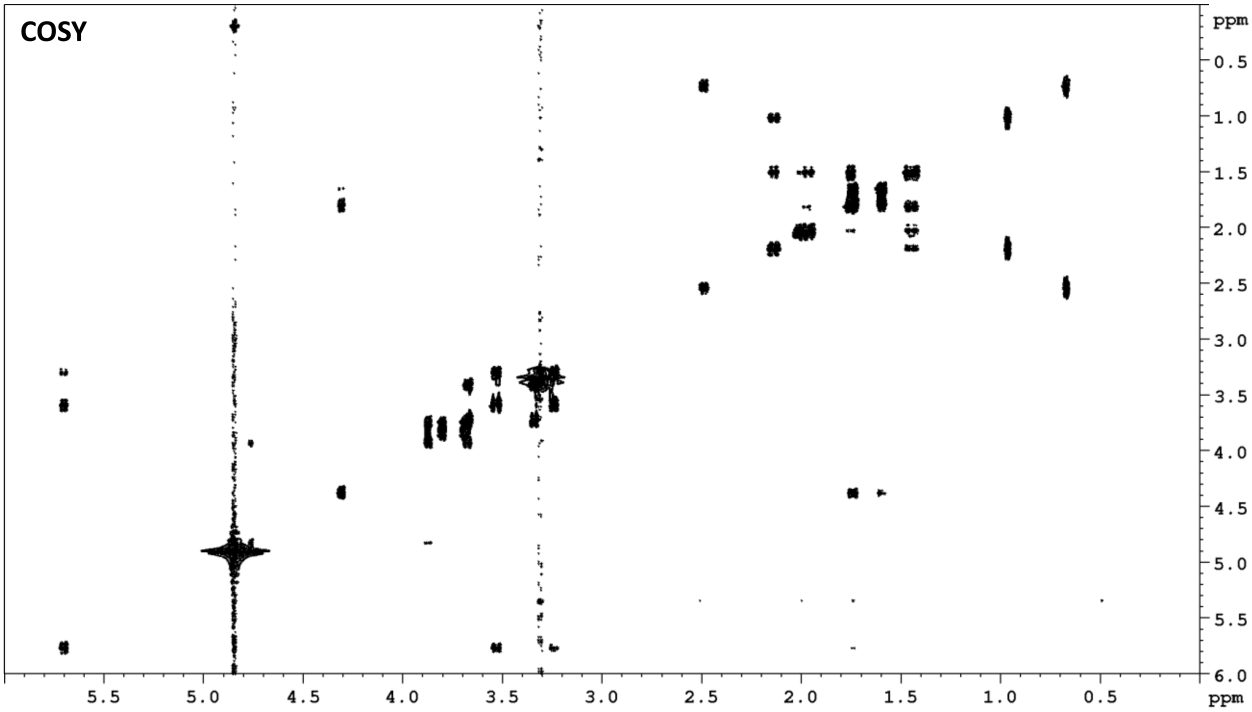

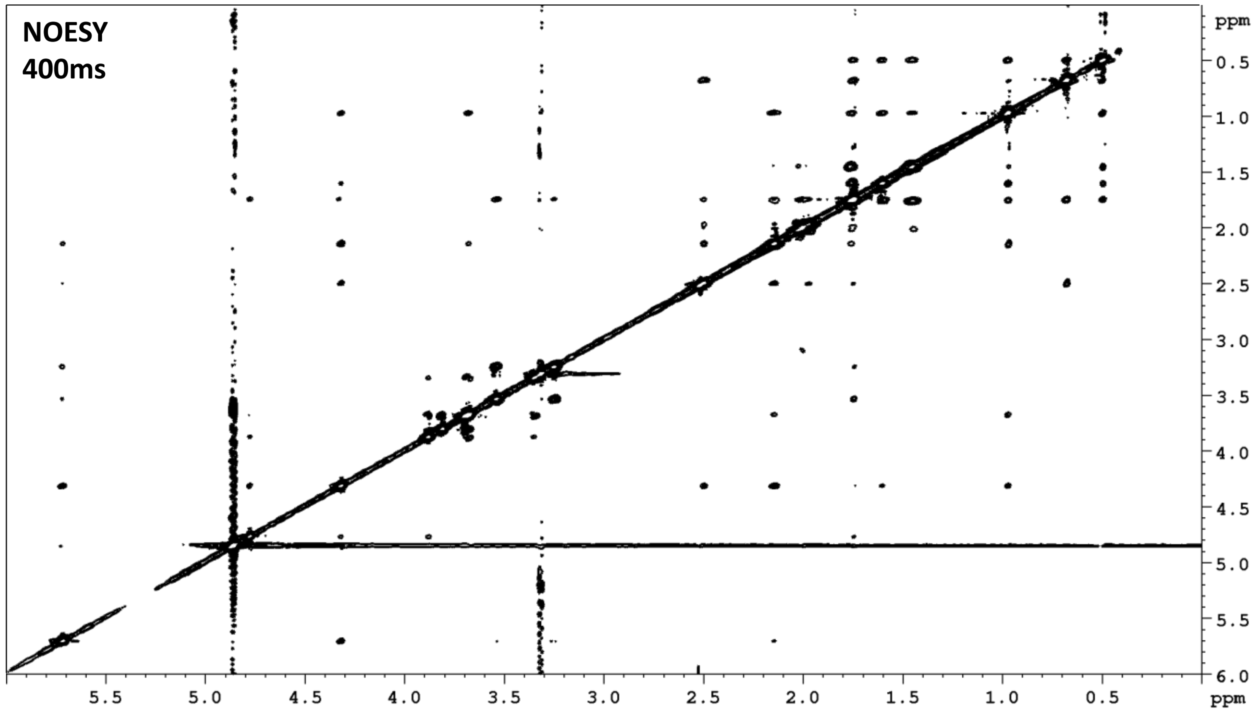
NOESY
400ms
ppm
0.5
1.0
1.5
2.0
2.5
3.0
3.5
4.0
4.5
5.0
5.5
6.0
5.5
5.0
4.5
4.0
3.5
3.0
2.5
2.0
1.5
1.0
0.5
ppm

Figure S19. 1D and 2D NMR spectra for 4’-ketoascochlorin (**20**) in $CDCl_3$.

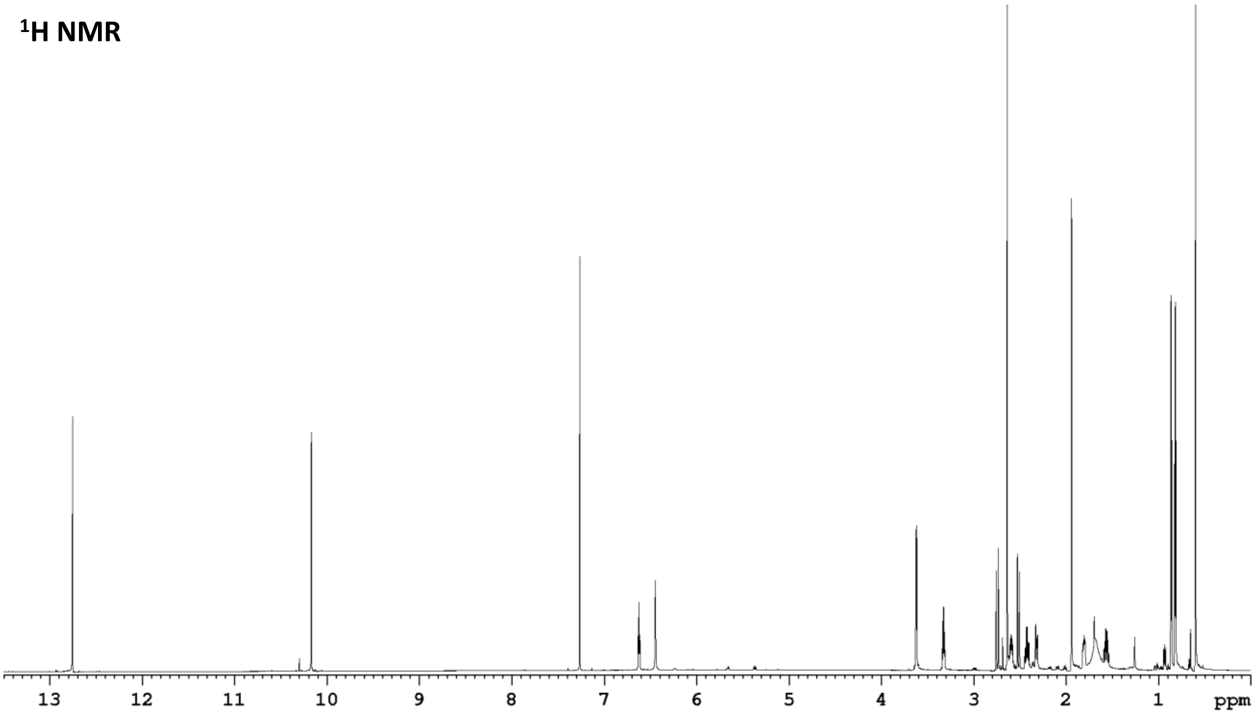

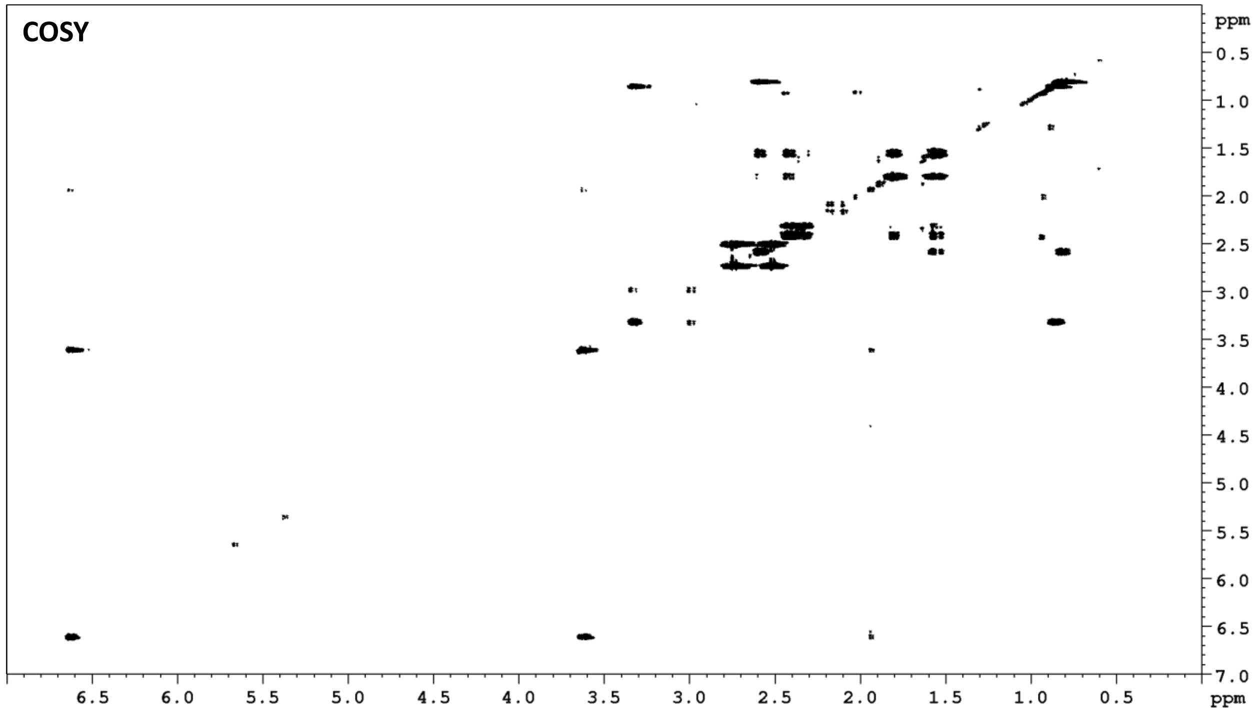

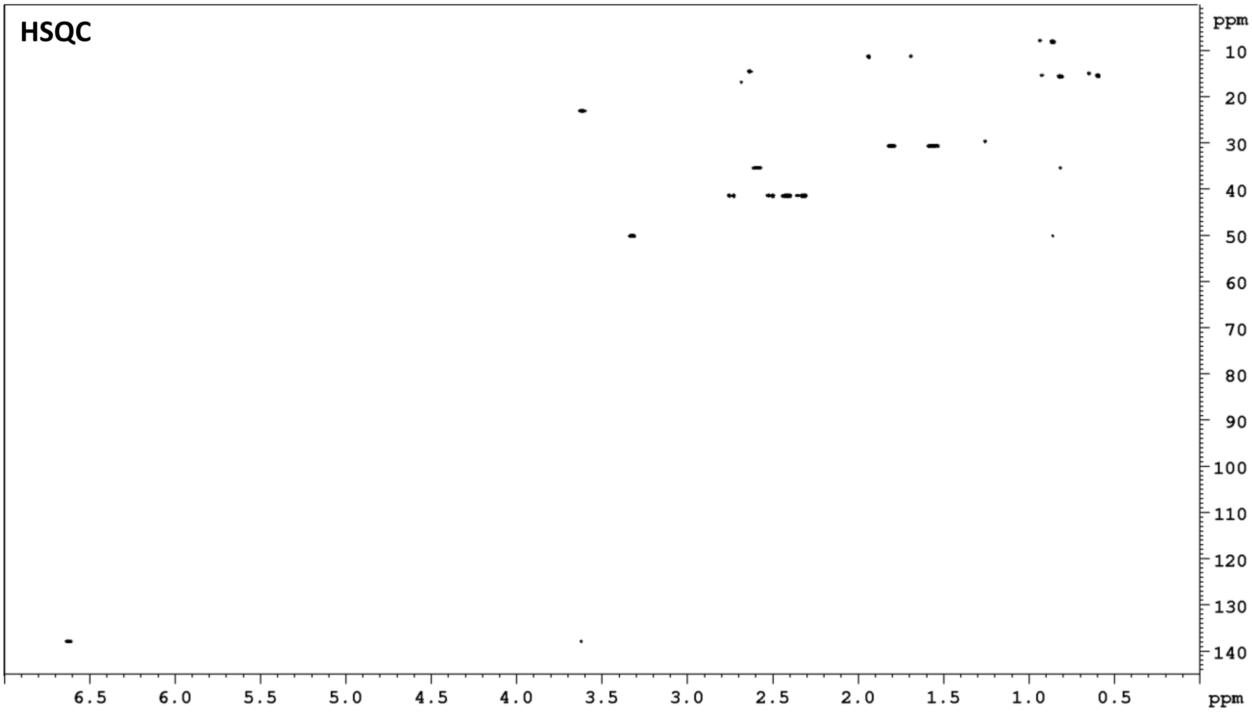

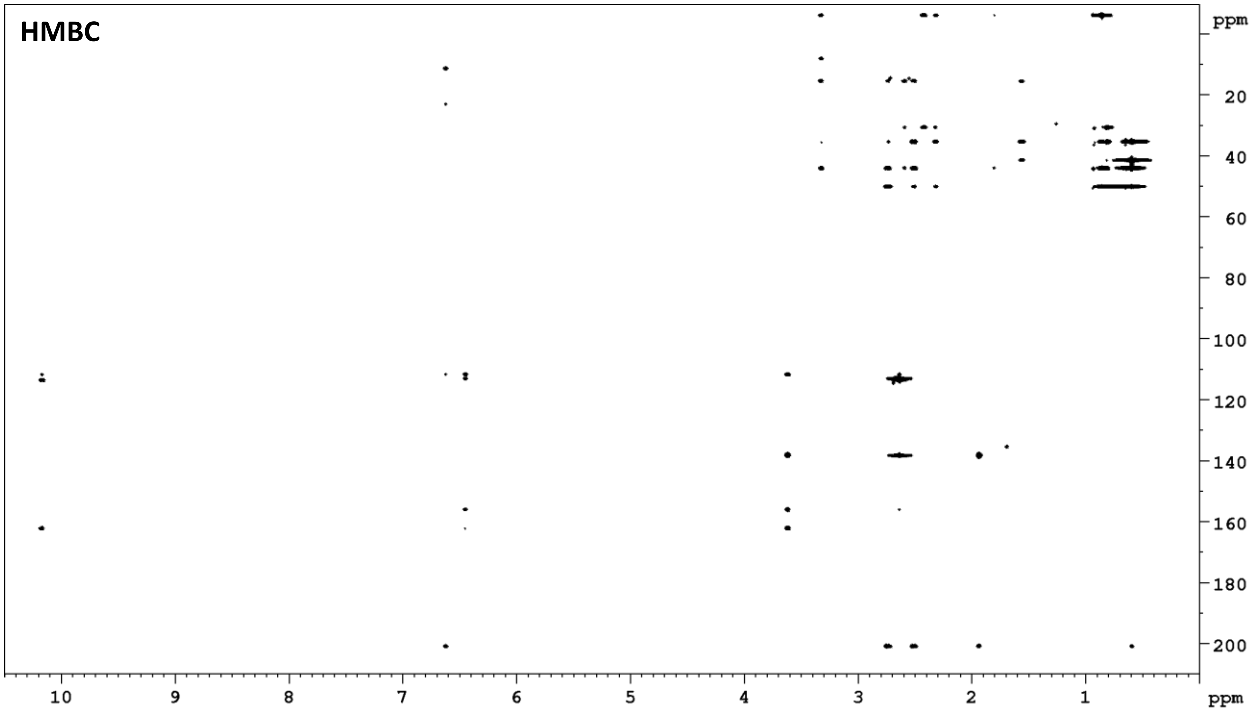

*The ketone group at C-10' is observed at the wrong ^{13}C chemical shift (δ_C -6.1ppm) due to ^{13}C SW set to 220ppm.

Figure S20. 1D and 2D NMR spectra for 4',5'- dehydro-4'-formylascochlorin (**21**) in $CDCl_3$.

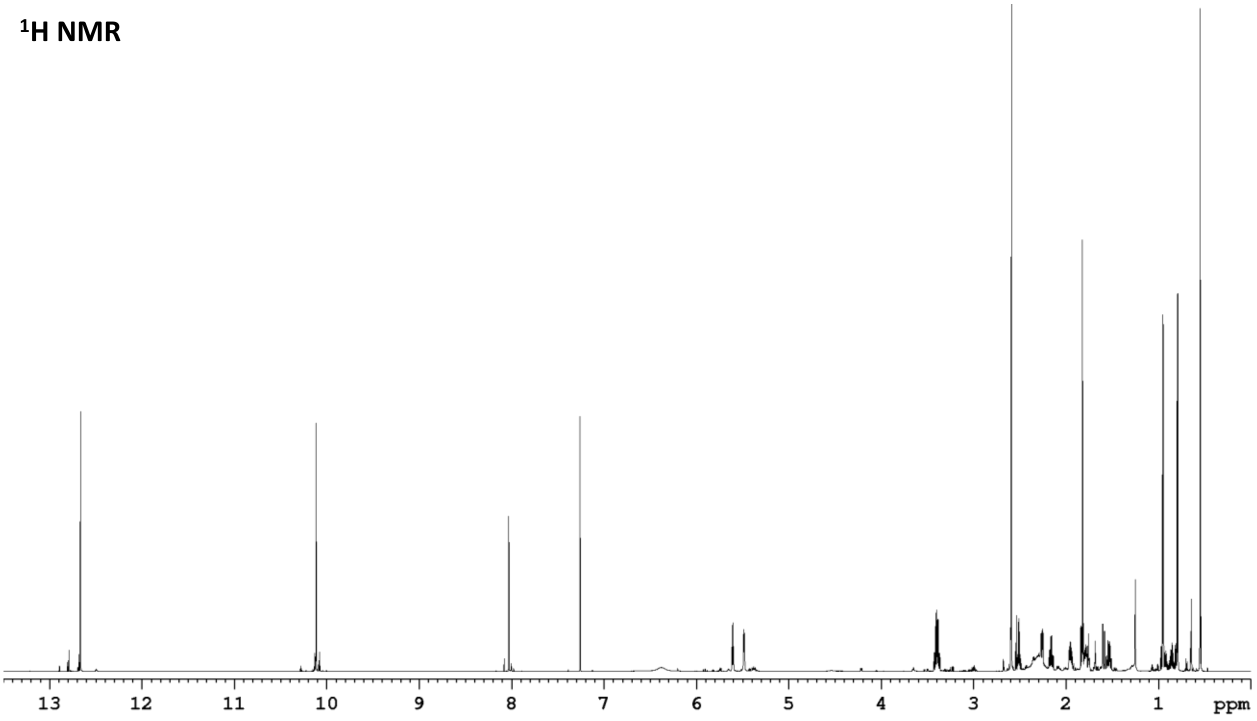

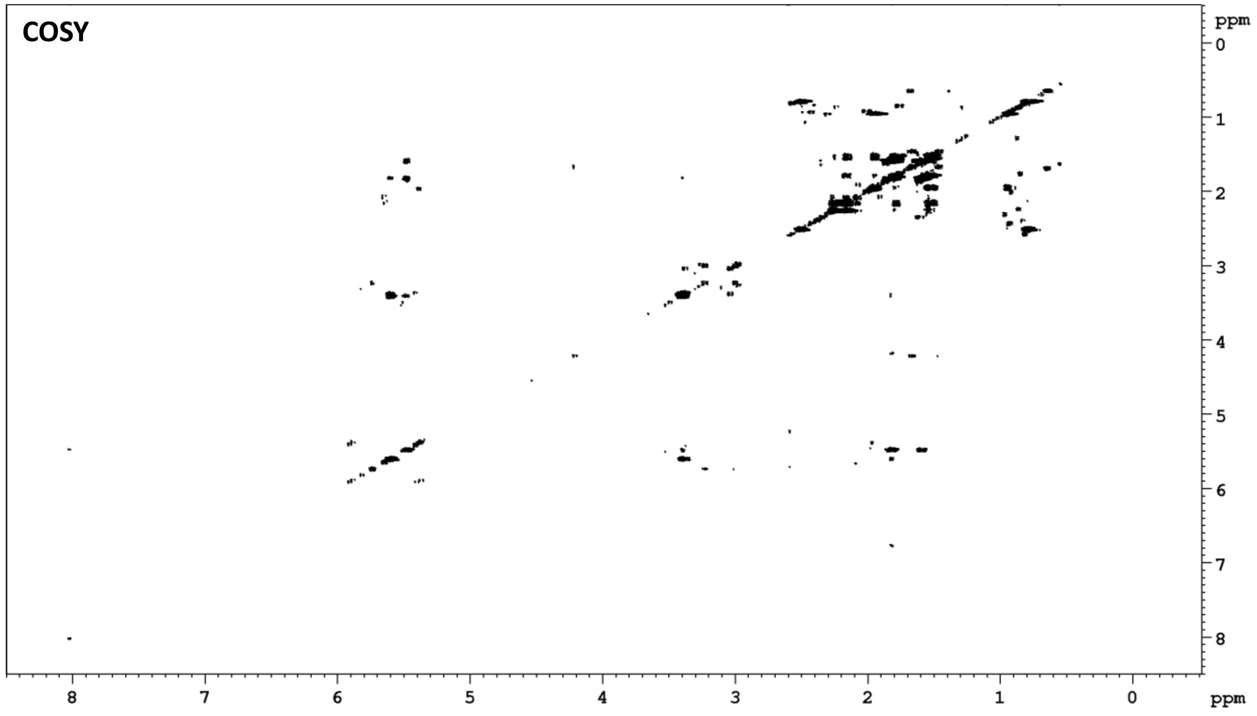

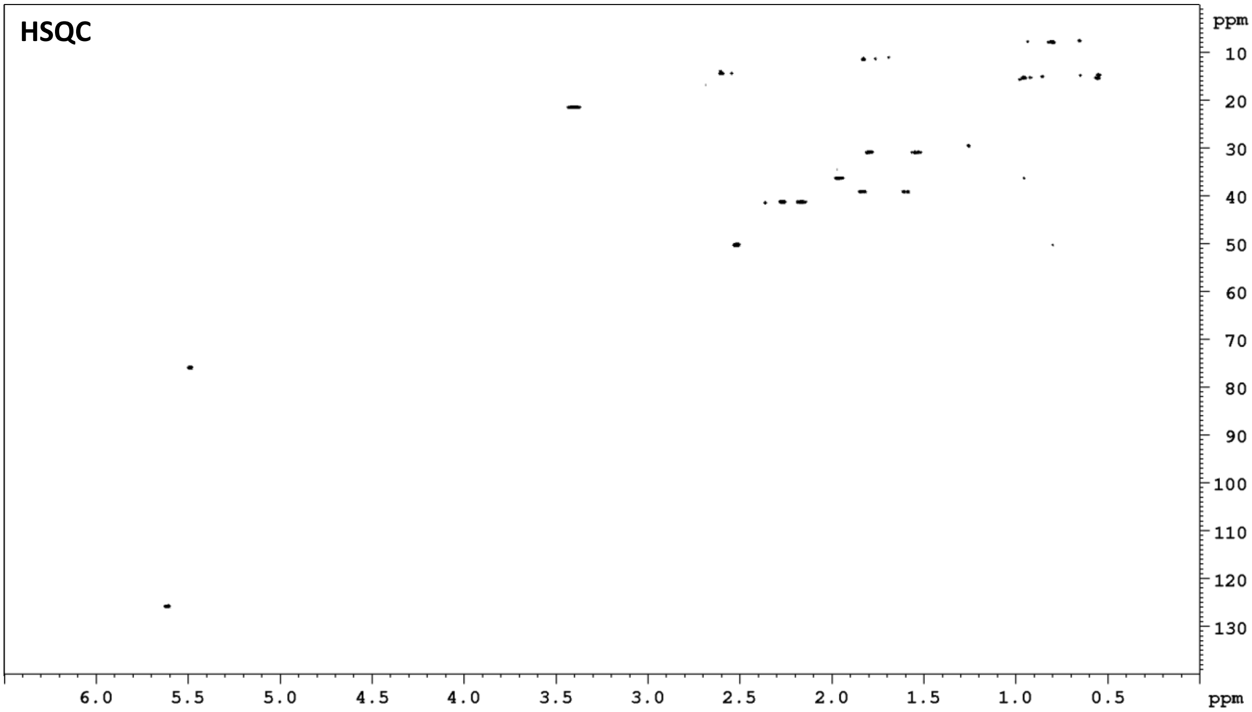

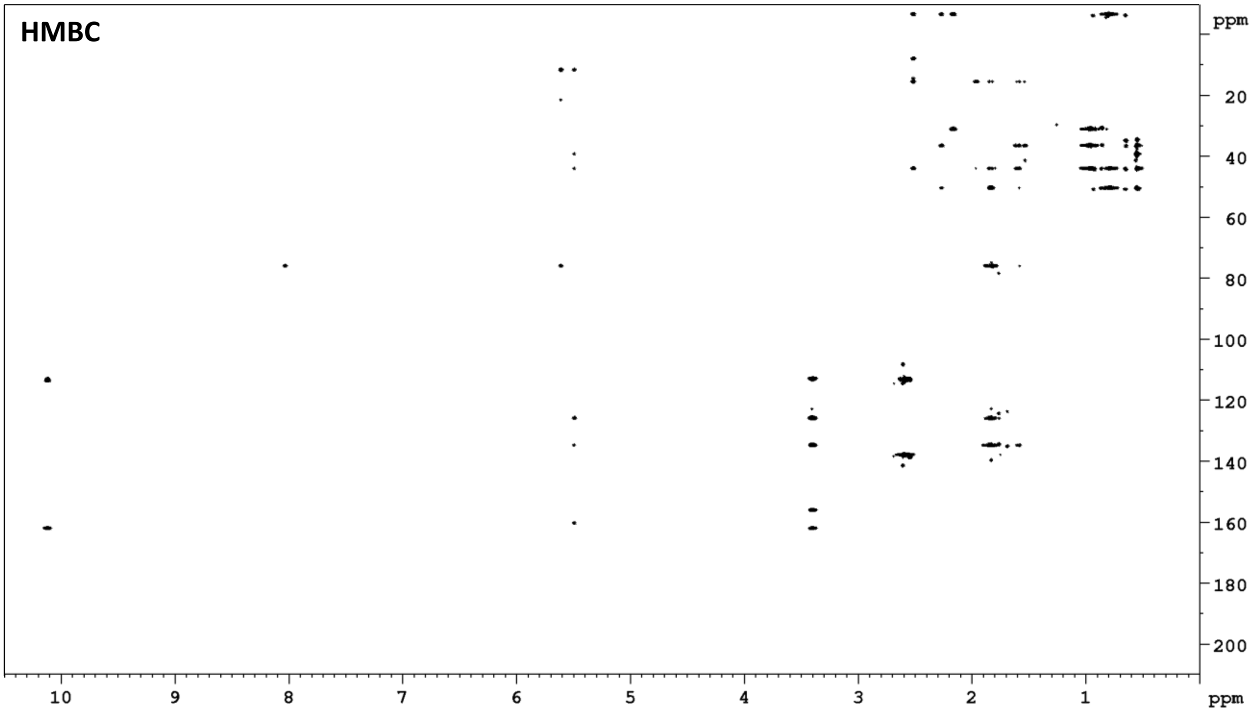

*The ketone group at C-10' is observed at the wrong ^{13}C chemical shift (δ_C -6.7ppm) due to ^{13}C SW set to 220ppm.

Figure S21. 1D and 2D NMR spectra for fimetarin A (**22**) in CD_3OD.

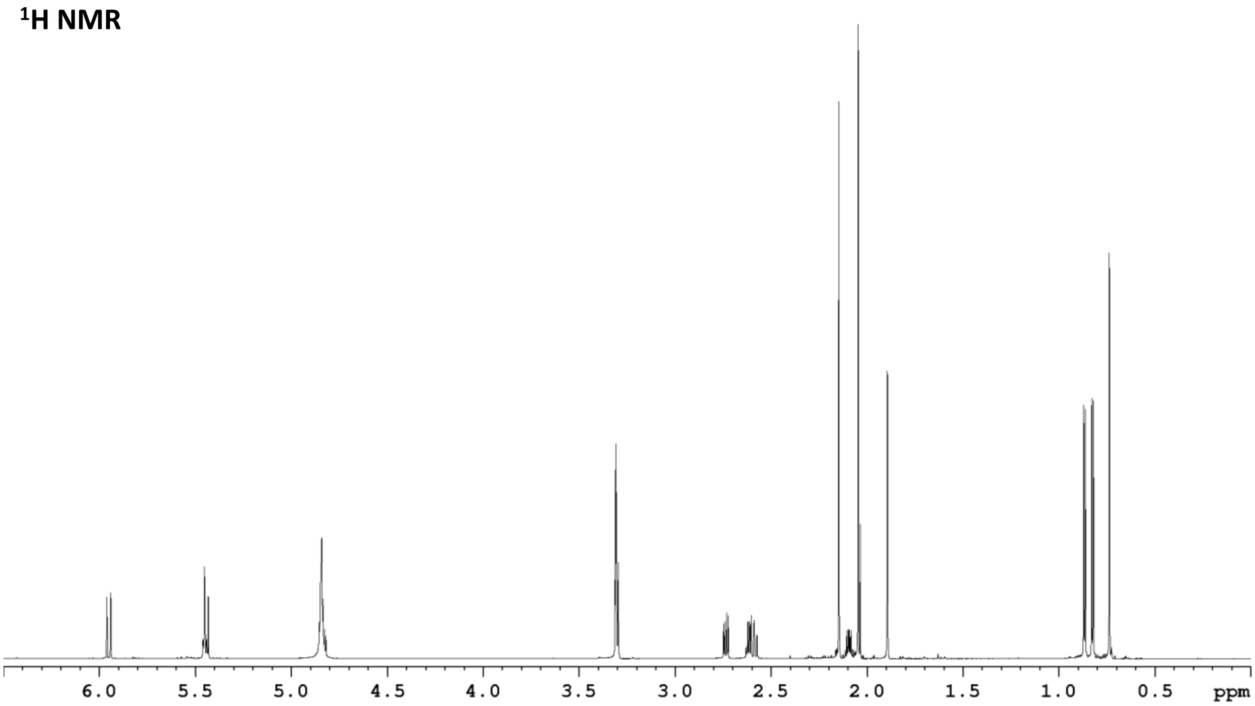

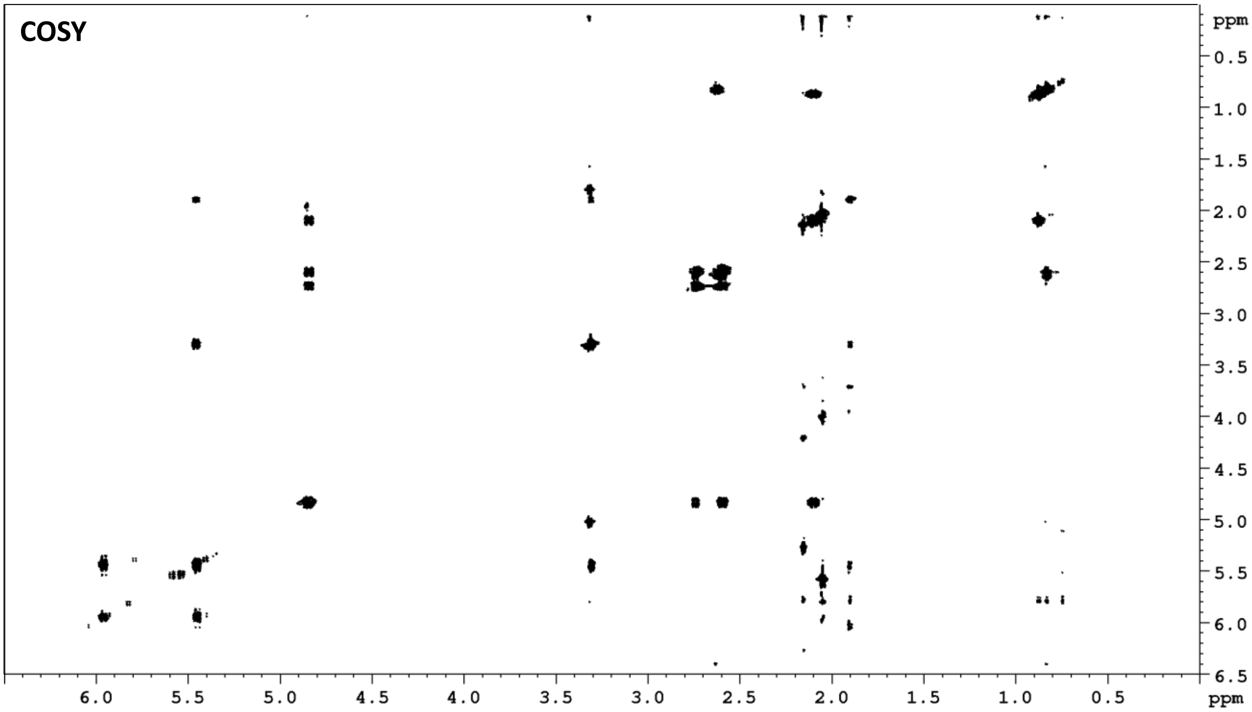

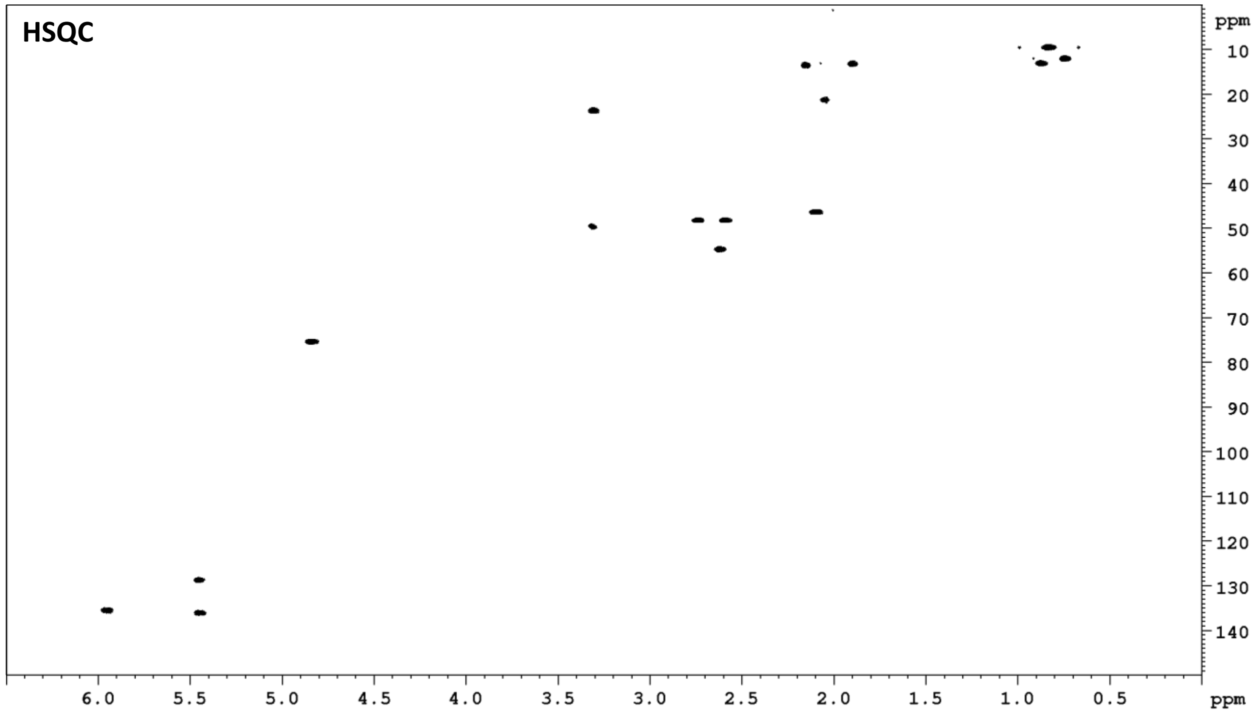
HSQC
ppm
10
20
30
40
50
60
70
80
90
100
110
120
130
140
6.0
5.5
5.0
4.5
4.0
3.5
3.0
2.5
2.0
1.5
1.0
0.5
ppm

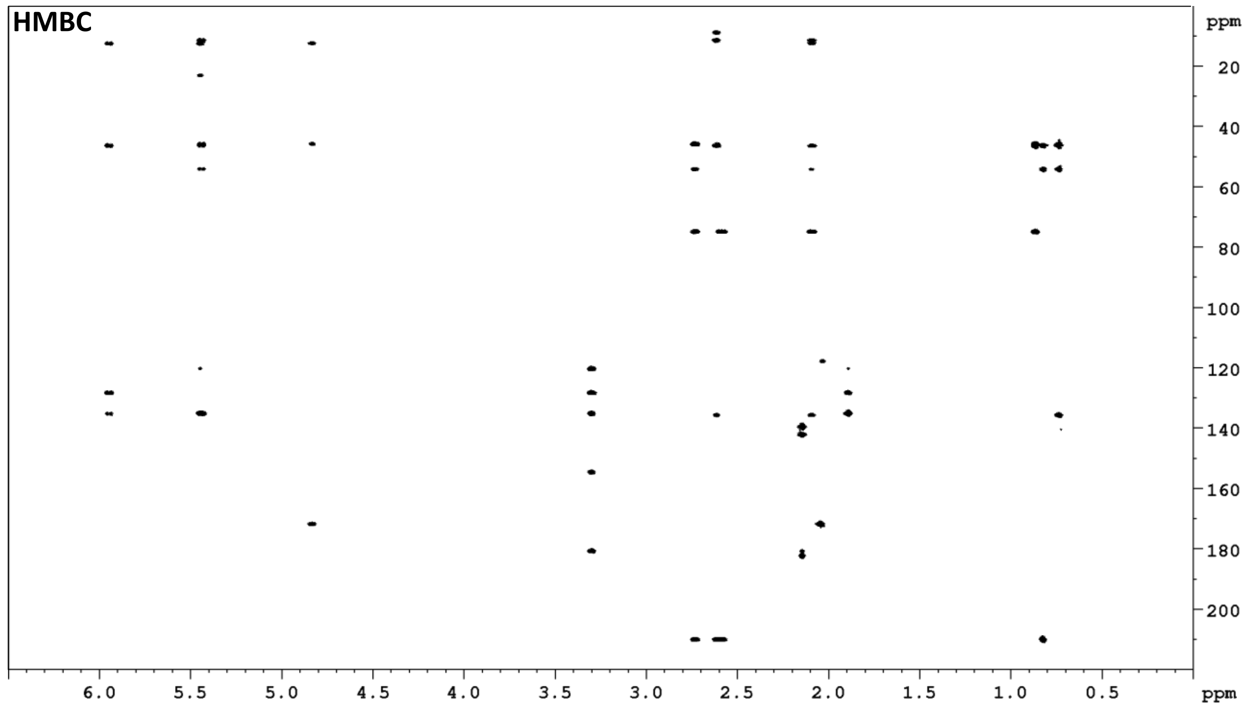
HMBC
ppm
20
40
60
80
100
120
140
160
180
200
6.0
5.5
5.0
4.5
4.0
3.5
3.0
2.5
2.0
1.5
1.0
0.5
ppm

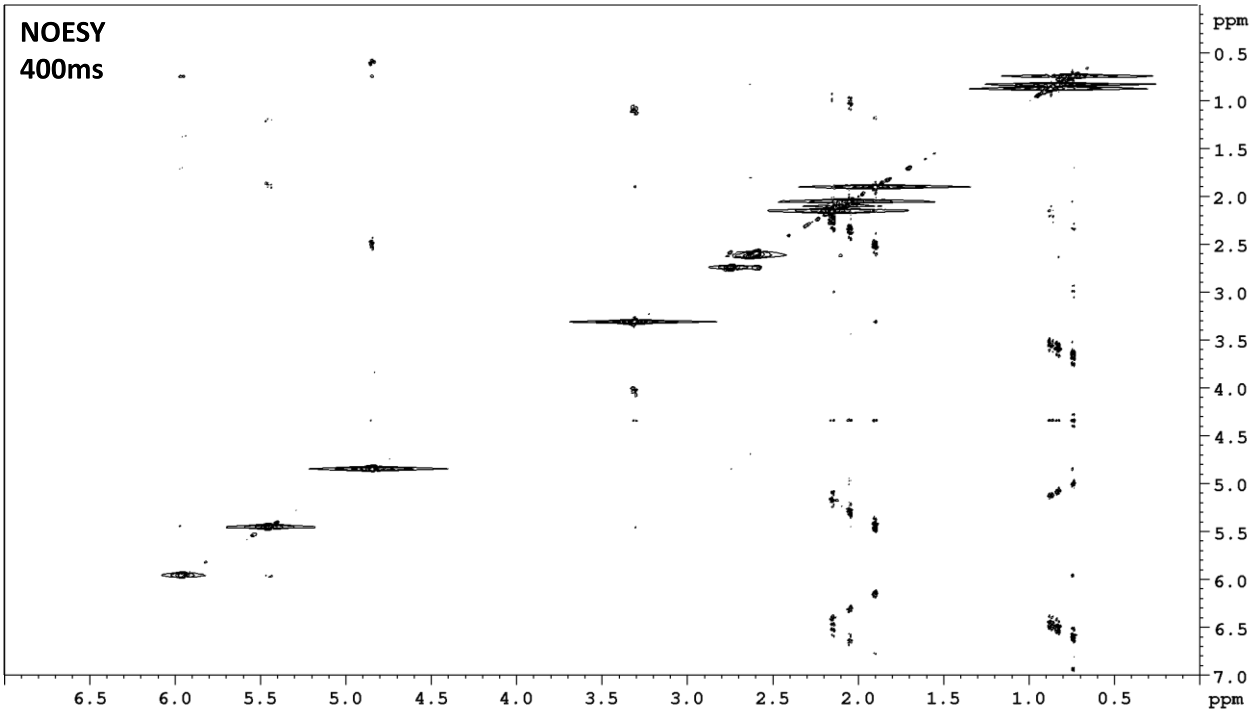
NOESY
400ms
ppm
0.5
1.0
1.5
2.0
2.5
3.0
3.5
4.0
4.5
5.0
5.5
6.0
6.5
7.0
6.5 6.0 5.5 5.0 4.5 4.0 3.5 3.0 2.5 2.0 1.5 1.0 0.5
ppm
